# Three New Endemic Species of Namib Day Geckos (Gekkonidae: *Rhoptropus*) From the Namibe Province, Angola

**DOI:** 10.1002/ece3.71609

**Published:** 2025-06-27

**Authors:** Javier Lobón‐Rovira, Matthew P. Heinicke, Aaron M. Bauer, Werner Conradie, Pedro Vaz Pinto

**Affiliations:** ^1^ CIBIO, Centro de Investigação em Biodiversidade e Recursos Genéticos, InBIO Laboratório Associado Universidade do Porto Vairão Portugal; ^2^ BIOPOLIS Program in Genomics, Biodiversity and Land Planning CIBIO Vairão Portugal; ^3^ Department of Natural Sciences University of Michigan‐Dearborn Dearborn Michigan USA; ^4^ Department of Biology and Center for Biodiversity and Ecosystem Stewardship Villanova University Villanova Pennsylvania USA; ^5^ Port Elizabeth Museum Gqeberha South Africa; ^6^ Department of Conservation Management, Natural Resource Science and Management Cluster, Faculty of Science Nelson Mandela University George South Africa; ^7^ TwinLab CIBIO/ISCED Instituto Superior de Ciências da Educação da Huíla Lubango Angola; ^8^ Fundação Kissama Luanda Angola

**Keywords:** Africa, endemism, Gekkonidae, Namib desert, *Rhoptropus crypticus* sp. nov., *Rhoptropus megocellus* sp. nov., *Rhoptropus minimus* sp. nov.

## Abstract

Angola remains one of the least explored countries in Africa, and several groups of reptiles still require taxonomic and phylogenetic revision. To shed light on the true diversity of geckos in this gecko diversity hotspot of southwestern Africa, we conducted fieldwork in some of the less explored coastal regions of southern Angola. As a result, we identified previously unknown populations of Namib day geckos (genus *Rhoptropus*) which represent candidate new species. Through a comprehensive revision of the group, we describe three new endemic *Rhoptropus* spp. from the northern region of Namibe Province in Angola, based on morphological, phylogenetic and biogeographic data: *R. minimus* sp. nov., *R. megocellus* sp. nov., and *R. crypticus* sp. nov. These findings contribute to a better understanding of the biogeographic patterns of gecko diversity in Angola, highlighting the importance of this region as a significant center of endemism and diversification in Africa.

## Graphical abstract

In this work, we described three new endemic species of Namib Day geckos (*Rhoptropus*) from the northern Benguela Province, highlighting the importance of this region as an important center of endemism.

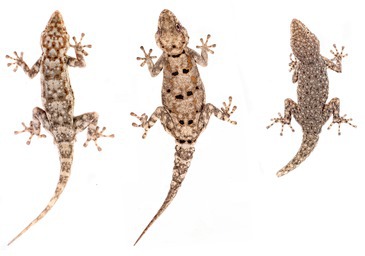

## 1 Introduction

Angola has recently emerged as one of the most gecko‐diverse countries in mainland Africa, ranking third after South Africa and Namibia, with 53 described species (Uetz et al. 2025; Röll et al. 2024; Parrinha et al. 2025) and several additional undescribed taxa already recognized. Notably, the number of known species has nearly doubled in the past few years (Marques et al. 2018; Branch et al. 2019). This rapid expansion of knowledge about Angolan herpetofauna is largely driven by improved access to previously unexplored areas, along with the application of phylogenetic techniques that have facilitated the identification of cryptic and previously overlooked species (Lobón‐Rovira 2023). As a result, several new species from historically remote regions such as Serra da Neve in Namibe Province, Mayombe National Park in Cabinda Province, and the Central Highlands have been described in the last five years (Marques et al. 2020; Branch et al. 2021; Conradie et al. 2022; Röll et al. 2024; Parrinha et al. 2025). Nevertheless, several species remain undescribed and are awaiting formal description (Lobón‐Rovira et al. 2022; Parrinha et al. 2025).

*Rhoptropus* is a unique genus of strictly rupicolous day geckos, endemic to the arid southwestern regions of Namibia and Angola (Bauer and Good 1996; Kuhn 2016) and the central highlands of Angola (Kuhn 2016; Parrinha et al. 2025). Surprisingly, this remarkable radiation appears to have speciated through non‐adaptive radiation, with some species in sympatry lacking clear evidence of morphological change (Bauer and Good 1996). Thus, this genus is characterized by high morphological conservatism and significant geographic overlap among congeners (Bauer and Good 1996). This has led to frequent morphological misidentifications between species, which have hindered taxonomic advancements for several decades. Nevertheless, the Angolan highlands appear to have driven allopatric speciation, resulting in several endemic species and clear biogeographic segregation (Kuhn 2016; Parrinha et al. 2025). As a result, Angola hosts nine out of the 11 species currently recognized in this genus (Kuhn 2016; Lobón‐Rovira et al. 2022, 2025; Parrinha et al. 2025). This includes four endemic species ( *R. montanus* Laurent 1964, *R. nivimontanus* Parrinha, Marques, Tuitenko, Heinicke, Bauer, Ceríaco 2025, *R. benguellensis* Mertens 1938, and *R. taeniostictus* Laurent 1964), and one potentially endemic undescribed taxon (*R*. aff. *barnardi*; Kuhn 2016). Not surprisingly, the phylogenetic history of this genus remains poorly resolved, as does its true diversity (Parrinha et al. 2025). Consequently, additional cryptic taxa may still be discovered, and further research is needed to clarify several unresolved evolutionary questions.

Therefore, to better understand the role of southwestern Angola in the diversification, speciation, and evolution of this still poorly understood genus of diurnal geckos and to better understand the true taxonomic diversity of this group and identify primary regions that have driven diversification in this group, surveys were conducted across southwestern Angola. As a result, we detected some morphologically distinctive *Rhoptropus* populations in the arid coastal region of the Namib Province that could not be assigned to any previously known species. Therefore, we used phylogenetic, morphological, and geographical data to assess their taxonomic status and to provide an updated phylogenetic hypothesis for this group.

## 2 Materials and Methods

### 2.1 Sampling

Between 2018 and February 2025, extensive herpetological surveys were conducted across southwestern Angola, specifically targeting *Rhoptropus* species. These efforts led to the detection and collection of 14 specimens and some additional tissue samples from previously unknown populations of *Rhoptropus* (Table [Link], [Link]) that could not be assigned to any previously described species from Angola. Selected specimens were euthanized by injection of tricaine methanesulfonate (MS222; American Veterinary Medical Association 2020). Afterward, the vouchers were fixed in either 10% formalin or 96% ethanol and thereafter transferred to 70% ethanol for permanent storage. Liver or muscle tissue was collected before fixation in 10% formalin and preserved in 96% ethanol. Vouchers were deposited in the Fundação Kissama‐Holísticos collection (FKH, Luanda, Angola), Museu de História Natural e da Ciência—Universidade do Porto (MHNC‐UP, Porto, Portugal) and the Museo Nacional de Ciencias Naturales (MNCN, Madrid, Spain).

### 2.2 Molecular Analyses

#### 2.2.1 Phylogenetic Analysis and Divergence Time

To provide a phylogenetic framework for these new populations of *Rhoptropus*, we generated maximum likelihood (ML) and Bayesian inference (BI) multilocus phylogenies using the previously published sequences available in GenBank, supplemented with newly generated genetic information (Table S1). To this aim, DNA was extracted using the EasySpin Genomic DNA Tissue Kit (Citomed, Portugal), following the manufacturer's protocols. Concentrations for the PCR were: 5 μL PCR Master Mix, 0.4 μL of each primer, 3.2 μL H_2_O, and 1–3 μL DNA (DNA aliquot volumes were adjusted to extraction results). Partial fragments of two mitochondrial genes, namely a ribosomal gene (*16S* rRNA; 544 bp) and a fragment of a protein‐coding gene and adjacent tRNAs (*ND2*; 952 bp), plus one partial fragment of a nuclear protein‐coding gene (*RAG‐1*; 966 bp) were obtained for most of the tissue samples detailed in Table S1. Primer and PCR reaction details are summarized in Table S2. The prepared PCR products were purified and sequenced at Centre for Molecular Analysis (CTM‐CIBIO, Porto, Portugal) and Macrogen Corp. (Amsterdam, Netherlands). Sequences were checked and edited using Geneious Prime v2024.0.5 (http://www.geneious.com/) and aligned using the MUSCLE plugin for Geneious. All sequences have been deposited in GenBank (Table S1).

Phylogenetic analyses were conducted on independent mitochondrial genes and a concatenated dataset of all three genes (*16S*, *ND2* and *RAG‐1*). Partitioning schemes were evaluated with PartitionFinder2 (Lanfear et al. 2012), and the optimal substitution model of sequence evolution was selected via ModelFinder in IQ‐Tree v2.3.4 (Minh et al. 2021) using the Bayesian Information Criterion (BIC). The dataset was partitioned by gene, as recommended by PartitionFinder2. The selected substitution models were TIM2 + F + I + G4 for *16S*, TN + F + I + G4 for *ND2*, and HKY + F + G4 for *RAG‐1*. ML analysis was performed in IQ‐Tree v2.3.4 (Trifinopoulos et al. 2016) using four partitions and 1000 bootstrap replicates with the ultrafast bootstrap approximation (UFBoot) method (Hoang et al. 2018). Bootstrap values of 95% or higher were considered to provide strong support (Huelsenbeck and Hillis 1993). For Bayesian Inference, MrBayes v3.2.7 (Ronquist et al. 2012) was used through CIPRES (Miller et al. 2010), with the dataset divided into four partitions. The BI analysis ran for 10 × 10^6^ generations using Metropolis‐coupled Markov chain Monte Carlo (MC^3^), sampling every 1000 generations. Convergence was evaluated by examining the effective sample size (ESS) values in Tracer v1.7 (Rambaut et al. 2014), ensuring all parameter ESS values exceeded 200. A 25% burn‐in was discarded, and a 50% majority‐rule consensus tree was generated in MrBayes. The substitution model space was set with the parameters *lset nst = mixed rates = invgamma*. Bootstrap support (BS) was assessed with 1000 pseudoreplicates, and posterior probabilities (PP) ≥ 0.95 were considered to indicate strong support. Finally, uncorrected pairwise sequence divergences (p‐distance) for *16S* and *ND2* were calculated in MEGA v10.1.7 (Kumar et al. 2018) to examine intra‐ and interspecific variations.

A comprehensive time calibrated Bayesian phylogeny for all species‐level lineages of *Rhoptropus* and other genera in the *Pachydactylus* group (*Chondrodactylus*, *Elasmodactylus*, *Pachydactylyus*) was estimated based on the data set and protocol from Heinicke et al. (2017), supplemented with recently published material from Marques et al. (2023) and Parrinha et al. (2025), additional *16S* sequences from Lamb and Bauer (2005) or sourced from GenBank, and newly generated sequences in this work (Table S3). The time‐tree data set includes 102 taxa and 3980 characters for the genes *16S*, *ND2* (and adjacent tRNAs), *KIF24*, *PDC*, and *RAG‐1*. The partitioning scheme and models of evolution used followed Heinicke et al. (2017) where applicable based on data overlap or were estimated with PartitionFinder2 otherwise, resulting in the following model selections: GTR + I + Γ for *16S*, *ND2* position 1, *ND2* position 2, *tRNA*; GTR + Γ for *ND2* position 3; TrN + I + Γ for *RAG‐1* position 1 & 2; HKY + Γ for *RAG‐hoh1* position 3, *KIF24* position 1 & 2; K80 + Γ for *PDC* position 3, *KIF24* position 3; TrNef + I + Γ for *PDC* position 1 & 2. The time‐tree analysis was conducted using BEAST v1.10.4 (Suchard et al. 2018), applying five gekkotan calibration priors following Heinicke et al. (2017). Two independent runs were conducted for 100 million generations each, with sampling every 10,000 generations and the first 10% of the runs (1000 samples per run) discarded as burn‐in. A maximum clade credibility tree with median node heights was generated using the remaining 18,000 post‐burn‐in samples from the two combined parallel runs. Tracer v1.7 (Rambaut et al. 2014) was used to evaluate parameter distributions across samples, with ESS values > 500 for all parameters used as the metric for determining adequate sampling.

#### 2.2.2 Haplotype Networks

Independent median‐joining haplotype networks were constructed for the relevant groups, namely the *R. barnardi* group, the *R. benguellensis* group, and the *R. taeniostictus* group. To this aim, phased nuclear alleles (*RAG‐1*) were generated in DnaSP v6 (Rozas et al. 2017), and the resulting phased sequences were used to generate median‐joining haplotype networks using Networks v4.6.1.1 (Bandelt et al. 1999), applying default settings and with a parsimony cut‐off of 95%.

### 2.3 Morphological Comparison

For the external morphological analyses, we examined a total of 44 specimens of *Rhoptropus* from Angola (Table S4). We recorded standardized morphometric measurements following Lobón‐Rovira et al. (2024) as follows: snout‐vent length (SVL, from tip of snout to anterior cloaca opening), tail length (TL, from tip of tail to posterior cloaca opening), head length (HL, from posterior end of occipital to tip of snout), head width (HW, at widest point), head height (HH, at highest point), snout to eye distance (SE, from tip of snout to anterior corner of eye), orbit diameter (OD), eye to eye distance (EE, from anterior corner of eye to anterior corner of eye), internarial distance (IN, shortest distance between nares), forearm length (FL, from elbow to base of the palm), hindlimb length (HindLL, from knee to heel), crus or tibia length (CL, from base of heel to knee), and trunk length (TrunkL, distance between inguinal and axillary regions). The meristic data collected were: number of supralabials, number of infralabials, chin shield arrangement, number of precloacal pores in males, and subdigital lamellae of the fourth toe from the base of the digits to the claw. All data were collected using a dissecting microscope and measurements were taken in millimeters (mm) with a digital caliper (accuracy of 0.1 mm).

## 3 Results

### 3.1 Molecular Analyses

**FIGURE 1:**
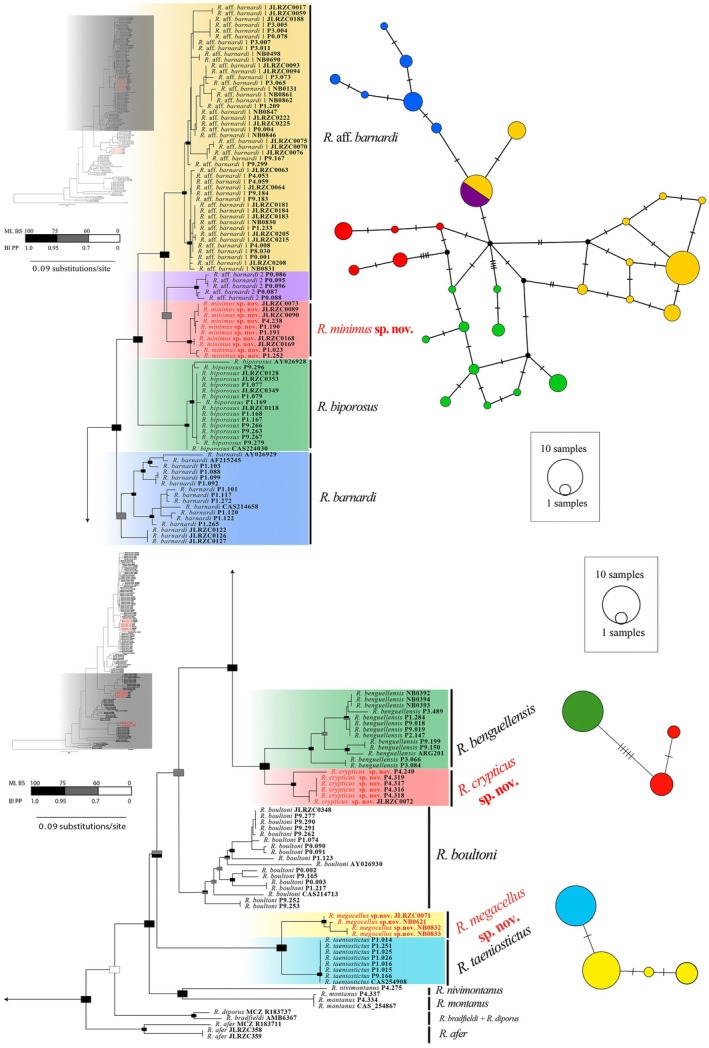
Maximum likelihood (ML) phylogenetic tree for Angolan *Rhoptropus* based on concatenated dataset, with Bayesian inference (BI) support overlaid. Support values (BI PP, Bayesian inference posterior probabilities; ML BS, maximum likelihood bootstrap values) are shown graphically at the nodes according to the colors shown in the inset key. On the right, median‐joining nuclear (*RAG‐1*) networks, for the respective *Rhoptropus* group. Colors indicate different mitochondrial lineages.

Both phylogenetic analyses (ML and BI) retrieved the same topology, whether using individual mitochondrial genes independently or the full concatenated dataset (i.e., mitochondrial and nuclear genes, see Figures [Link], [Link]). However, they showed different support values for some nodes (Figure 1), lacking internal nodal support in some cases, as found in previous studies (Heinicke et al. 2017; Parrinha et al. 2025). The phylogenetic analyses recovered 14 well‐supported clades, which consistently differ from one another by pairwise genetic distances (p‐distances) of 7.2%–26.9% in the *ND2* gene (Table 1) and between 3.5%–15.7% in the *16S* gene. Furthermore, these clades form discrete morphological units and also do not share *RAG‐1* haplotype (see below). Thus, we consider that the 14 clades may represent operational taxonomic units (OTUs) (Figure 1). Among the pairwise comparisons (Table 1), the lowest genetic distance is that between *Rhoptropus bradfieldi* and *R. diporus* (7.2% in *ND2* and 3.54% in *16S*).

**TABLE 1:** *ND2* divergences (uncorrected pairwise distances) between and within Angolan *Rhoptropus*.

Species	1	2	3	4	5	6	7	8	9	10	11	12	13	14	15
1. Outgroup	—														
2. *R. montanus*	34.35	0.00													
3. *R. bradfieldi*	31.51	23.70	—												
4. *R. diporus*	30.86	24.71	7.24	—											
5. *R. afer*	34.18	25.53	22.60	22.48	—										
6. *R. benguellensis*	34.17	24.81	22.81	23.73	25.59	6.47									
7. *R. crypticus* sp. nov.	33.67	24.24	21.86	21.75	25.05	16.16	—								
8. *R. boultoni*	31.20	21.56	19.99	19.34	22.44	19.74	18.23	9.71							
9. *R. biporosus*	35.79	26.99	23.65	23.41	24.66	21.29	19.02	19.57	2.17						
10. *R. barnardi*	34.43	25.68	22.81	22.63	23.43	19.58	17.32	18.26	14.64	—					
11. *R*. aff. *barnardi* 2	33.62	26.37	23.07	22.27	24.16	21.49	18.87	19.84	15.31	15.48	—				
12. *R*. aff. *barnardi* 1	31.74	25.80	21.88	20.64	22.84	19.95	17.08	18.90	14.95	16.92	10.47	2.42			
13. *R. taeniostictus*	34.29	25.55	24.37	25.02	26.52	23.78	22.68	21.75	26.02	22.58	22.76	22.85	0.087		
14. *R. megocellus* sp. nov.	33.90	25.58	22.47	22.24	26.99	22.61	23.34	21.74	26.18	23.83	23.69	23.53	13.35	3.38	
15. *R. minimus* sp. nov.	31.14	25.16	21.03	20.32	23.31	19.73	17.48	18.70	13.50	16.17	10.53	8.68	22.26	23.04	0.71

*Note:* Bold values depict intraspecific divergences. (Outgroup = *Chondrodactylus* spp.).

**FIGURE 2:**
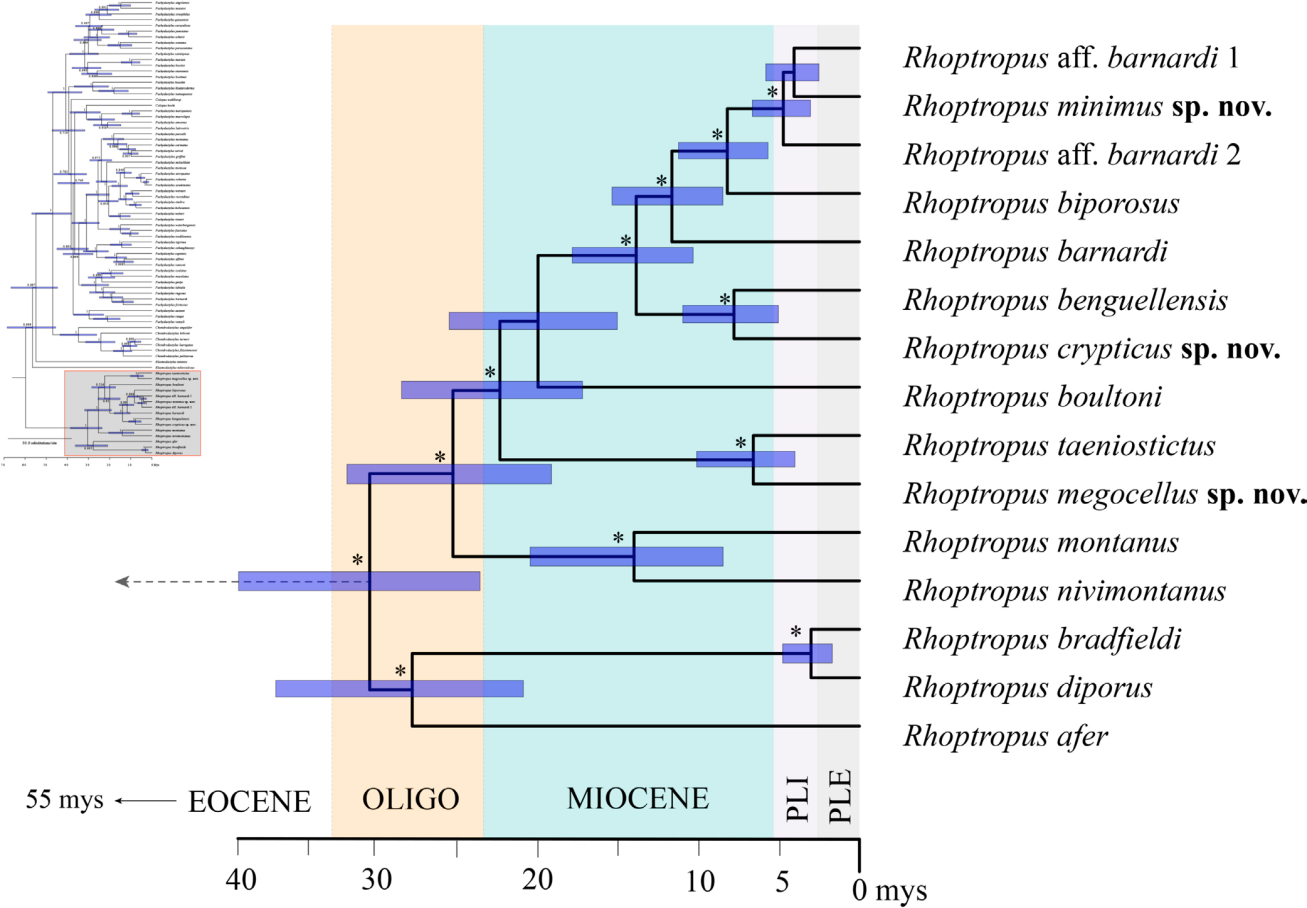
Detailed time‐calibrated species‐level Bayesian phylogeny among Namib Day Geckos (*Rhoptropus*). Branches with posterior probability > 0.9 are denoted by asterisks (*) at relevant nodes. Blue bars depict 95% Highest Posterior Density (HPD) intervals of estimated divergence dates. Full time‐calibrated species tree species in Figure S3. Mys, million years; OLI, Oligocene; PLE, Pleistocene; PLI, Pliocene.

The different phylogenetic reconstructions identified the split between *Rhoptropus afer* and all other members of the group as the most basal node (PP: 1.0, BS: 100%), which is in agreement with previous studies (Kuhn 2016; Parrinha et al. 2025). *Rhoptropus afer* differs from its congeners by a minimum uncorrected p‐distances of 22% for *ND2* and 9.86% for *16S*. This is followed by another split between the two Namibian endemic species, *R. bradfieldi* and *R. diporus* from all the other members of the group. However, although these results agreed with previously published phylogenetic hypotheses for the group (Kuhn 2016; Parrinha et al. 2025), the phylogenetic placement of this clade lacks statistical support (PP: 0.65, BS: 56.6%). Therefore, the relationship between these two taxa and the other members of the group should be considered with caution. It must be further noted, that *R. bradfieldi* and *R. diporus* also present the lowest genetic distances between different OTUs (e.g., ~7.2% *ND2* uncorrected p‐distance), which we regard as the baseline divergence between species. All the other members cluster together in a well‐supported large clade (PP: 0.98, BS: 98.7%). In contrast, it must be noted that the time‐calibrated tree recovers *R. afer* + *R. bradfieldi* + *R. diporus* as a well‐supported phylogenetic clade, which seems to diverge from all the other congeners between 36.2 and 20.8 million years (Mys) during the Oligocene (Figure 2) based on the time‐tree analysis herein.

The largest monophyletic subgroup (PP: 1.0, BS: 98.9%), is represented by all members of the *R. barnardi* group, including *R. barnardi* sensu stricto, *R. biporosus* , and three additional OTUs from Angola, which is estimated to diverge from other *Rhoptropus* species during the Mid‐Miocene [19.1–31.8 Mys] (Figure 2). These OTUs differ by minimum uncorrected p‐distances of 10% in *ND2* and 3.9% in *16S*, (Table 1), thus above the species threshold proposed for this group. This group clusters as sister to another Angolan endemic clade (PP: 1.0, BS: 100%) that includes one lineage restricted to the Angolan escarpment ( *R. benguellensis* ) and a second lineage from the coastal region of northern Namibe Province, Angola, which represent an additional OTU (Figure 1). The *ND2* and *16S* uncorrected p‐distances are larger than 16.0% and 7%, respectively, and they theoretically diverged from each other between the Mid‐Miocene and the Late Miocene [5–11.0 Mys] based on the time‐tree analysis.

Furthermore, the phylogenetic reconstructions recover an independent clade which includes two well‐differentiated mitochondrial lineages (PP: 1, BS: 100%), here ascribed as the *R. taeniostictus* group, the relationships of which to other members of the group remain unresolved (PP: 0.98, BS: 61%). Nevertheless, the species‐tree recovers a well‐supported clade (PP:1) which seems to have diverged from other members of the group during the Mid‐Miocene [17.2–28.4 Mys]. These two lineages differ by 13.35% in the *ND2* uncorrected p‐distance and 5.0% in the *16S*, and diverged from each other during the Late Miocene [4–10.1 Mys] (Figure 2). Finally, other independent lineages are present, each representing a nominal species, including *R. montanus* *+ R. nivimontanus* and *R. boultoni* (Figure 1) which represent clades that diverge during the Early Miocene (see Figure 2).

In addition, the median‐joining network for the *RAG‐1* nuclear marker of the *R. barnardi* group (Figure 1) recovered a total of 31 alleles, with all members showing similar mutational steps between them. Although the different alleles largely grouped the considered OTUs, we detected that *R*. aff. *barnardi* 1 (yellow) and *R*. aff. *barnardi* 2 (purple) shared nuclear haplotypes between them. Consequently, while the two OTUs represent two well‐differentiated mitochondrial lineages (10.5% *ND2* uncorrected p‐distance), allele sharing detected between them may suggest secondary contact between these two lineages. Alternatively, both lineages may represent the same OTU, reflecting the effects of incomplete lineage sorting due to ancient polymorphism. Remarkably, the median‐joining network did not detect any evidence of allele sharing between *R*. aff. *barnardi* 2 and the other sister OTU, which is morphologically more similar to *R. biporosus* . Finally, significant mutational steps were detected in *R. biporosus* (green) and the other lineage from the coastal region of Namibe Province (red), suggesting restricted and isolated distributions among populations of these two species. Finally, the median‐joining network for the *RAG1* nuclear marker separates all the different OTUs, without any evidence of sharing haplotypes between them (Figure 1).

### 3.2 Morphological Comparisons

The results of the molecular analysis support the presence of four undescribed OTUs within Angolan territory, based on the phylogenetic hypothesis, genetic distance, and lack of haplotype sharing with other closely related species (see above). Despite the high morphological conservatism of this group, the four undescribed OTUs exhibit some diagnosable characters distinguishing them from their closely related congeners, based on coloration, scalation, and meristic data (see species accounts). Among them, *R*. aff. *barnardi* was previously assessed by Kuhn (2016) and is being described as a separate species in another study (Parrinha et al. in prep.). We infer that the other three OTUs, one sister to *R. benguellensis* , one sister to *R. taeniostictus* , and one new taxon within the *R. barnardi* group, represent independently evolving lineages (de Queiroz 2007) because they are well supported by congruent molecular, morphological, and biogeographic/ecological evidence. Consequently, three new Angolan endemic species of *Rhoptropus* are described herein.

**FIGURE 3:**
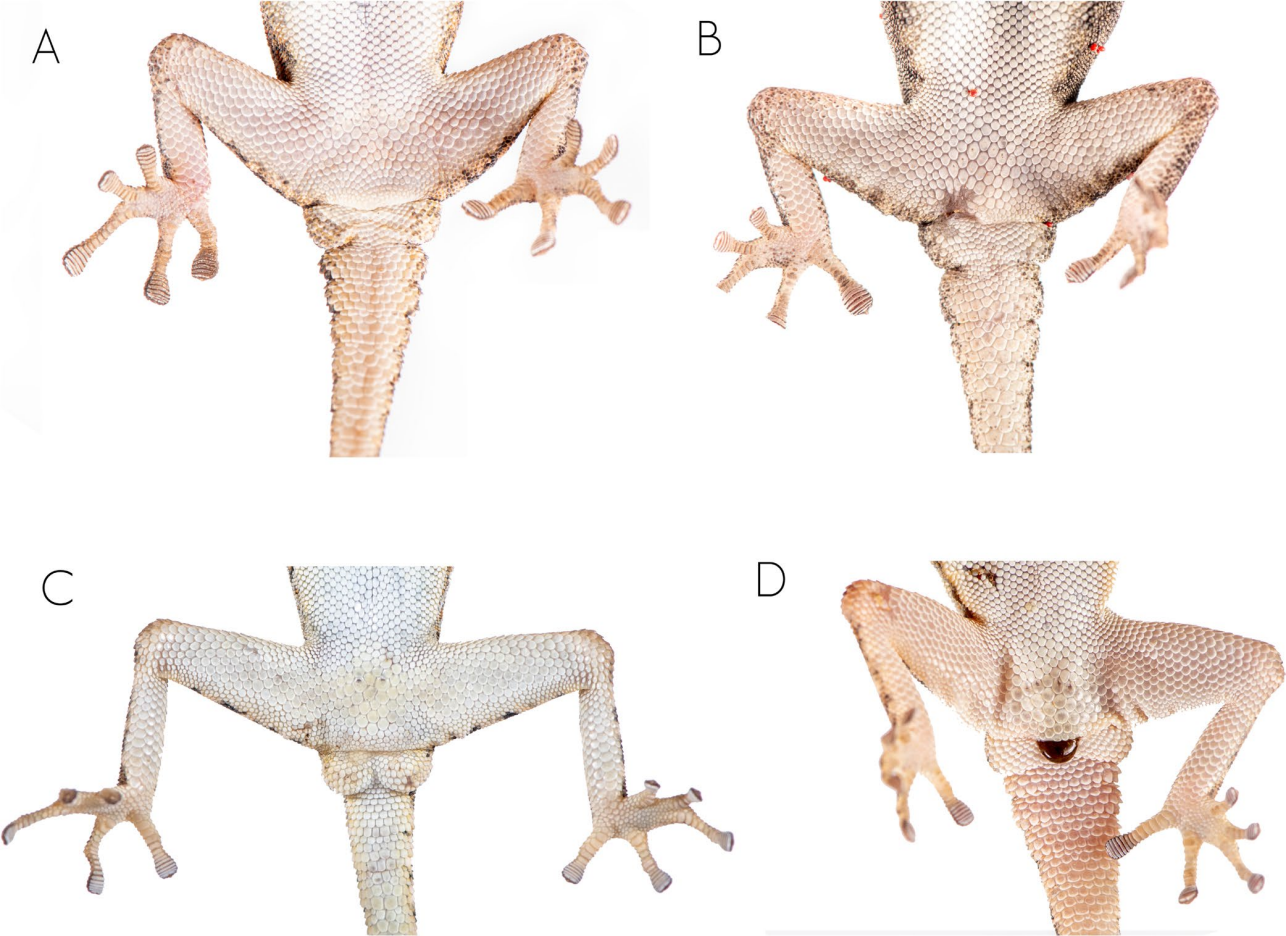
Detail of the cloacal region of member of the *Rhoptropus barnardi* group in Angola. (A) *Rhoptropus barnardi* sensu stricto from Calueque, Cunene Province, Angola. (B) *Rhoptropus* aff. *barnardi* 1 from Tchivira, Huila Province. (C) *Rhoptropus biporosus* from Iona National Park, Namibe Province, Angola. (D) *Rhoptropus minimus* sp. nov. from Salodjamba, Iona National Park entrance, Namibe Province.

**FIGURE 4:**
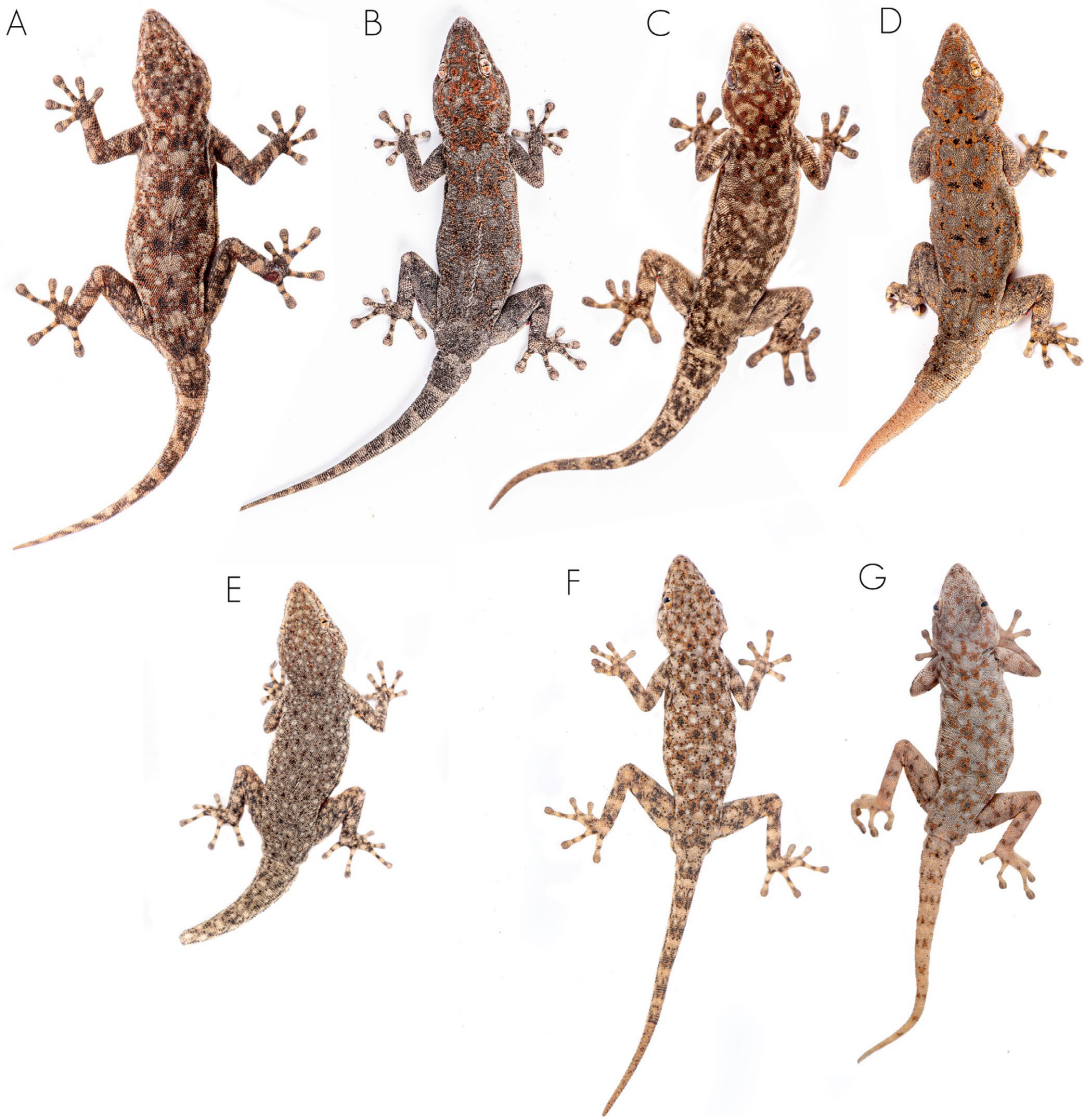
Unscaled photographs of the dorsal color pattern variation of the members of the *Rhoptropus barnardi* group in Angola. (A) *Rhoptropus barnardi* sensu stricto from Calueque, Cunene Province, Angola. (B) *Rhoptropus* aff. *barnardi* 2 from Bero River, Cunene Province. (C, D) *Rhoptropus* aff. *barnardi* 1 from Tchivira, Huila Province, and from Chapeu Armado, Namibe Province, Angola, respectively. (E) *Rhoptropus minimus* sp. nov. from Baba, Namibe Province. (F, G) *Rhoptropus biporosus* from Iona National Park, Namibe Province, Angola.

**FIGURE 5:**
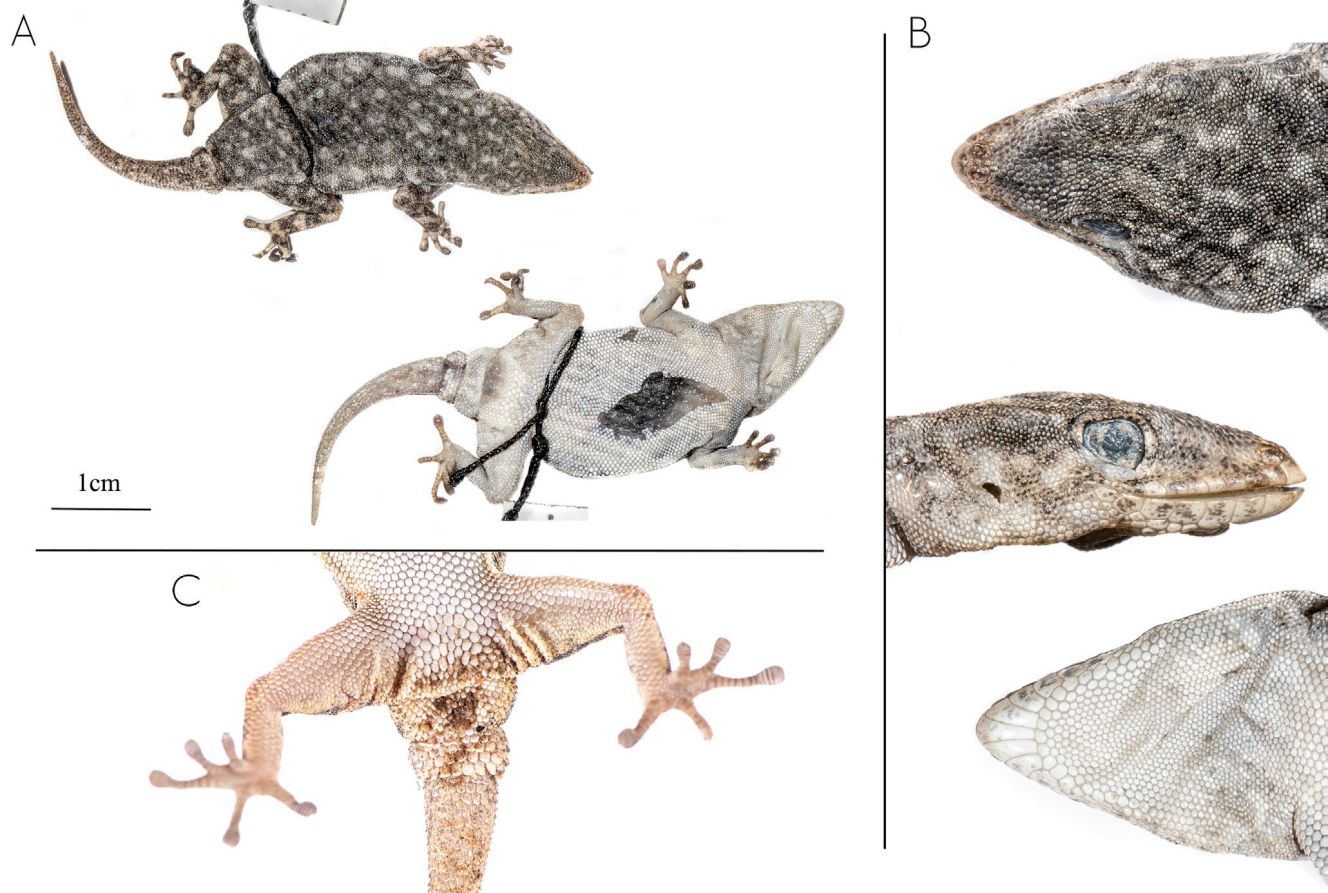
Holotype of *Rhoptropus minimus* sp. nov. (MNCN53501; Field Number P4.238) from Bentiaba, Namibe Province, Angola. (A) Photographs of the full body in dorsal (above) and ventral (below) views. (B) Details of the head in dorsal, lateral and ventral views. (C) Detail of the feet and subcaudals.

**FIGURE 6:**
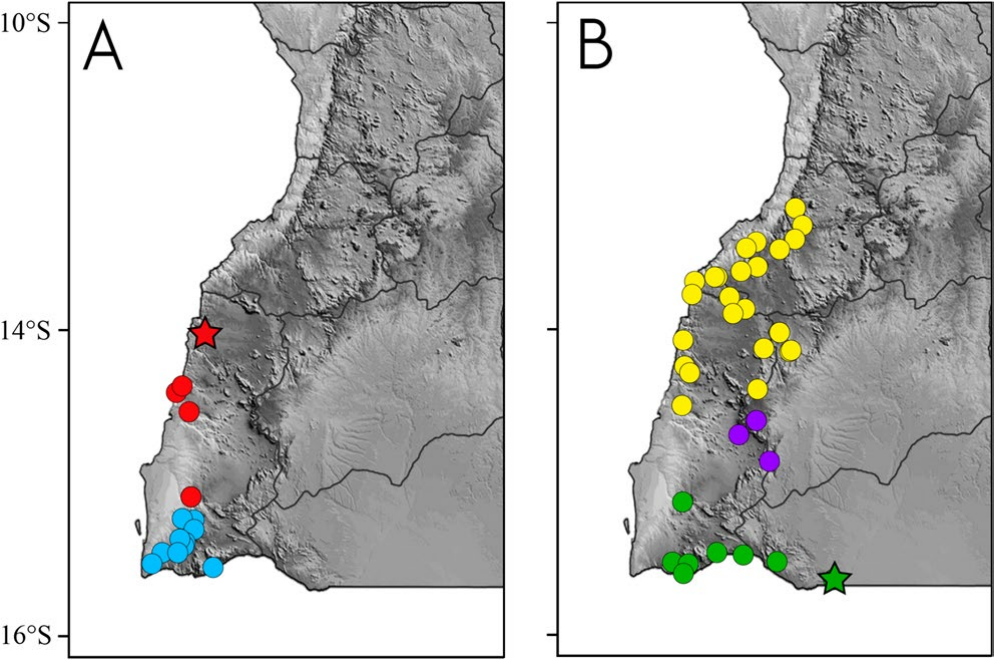
Distribution of the members of the *Rhoptropus barnardi* group in Angola. (A) Distribution of *R. minimus* sp. nov. (red) and *R. biporosus* (blue). (B) Distribution of *R. barnardi* sensu stricto (green), *R*. aff. *barnardi* 1 (yellow) and *R*. aff. *barnardi* 2 (purple). Stars depict the type localities.

**FIGURE 7:**
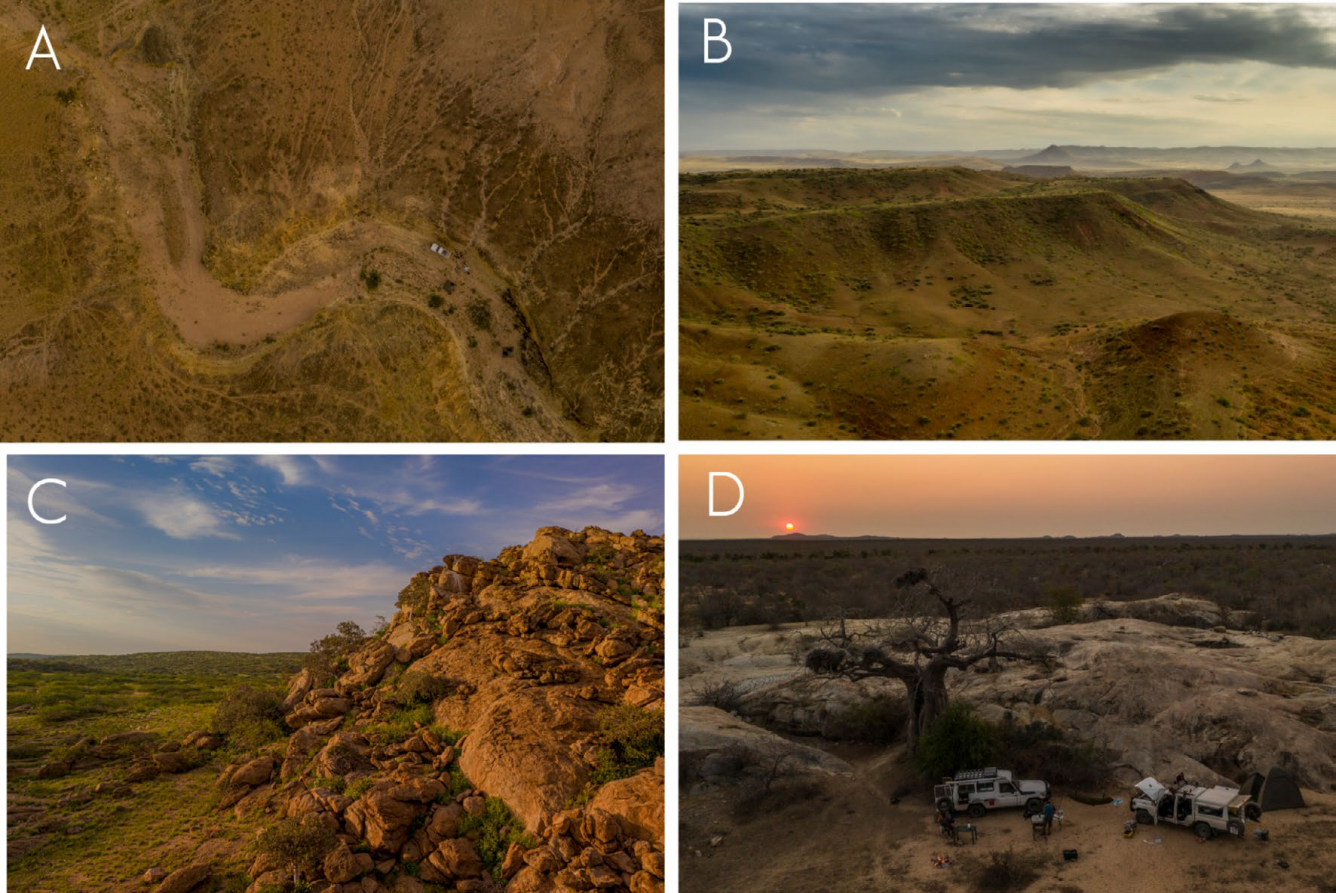
Photographs of (A, B) the habitat of *Rhotropus minimus* sp. nov. at Baba region and (C, D) habitat of *R*. aff. *barnardi* 1 at Meva and near Chicambi, Namibe Province, Angola.

***Rhoptropus minimus* sp. nov**.

https://zoobank.org/5F1E4A26‐E62C‐4984‐913A‐E08EF5193659

(Figures 3, 4, 5, 6, 7, Table 2 and Table S4)

**TABLE 2:** Means of the morphological (morphometric and meristic) of species closely related to *R. minimus* sp. nov. from the *Rhoptropus barnardi* group.

Species	*R. minimus* sp. nov.	*Rhoptropus biporosus*	*R*. aff. *barnardi*
	*n* = 5	*n* = 14	*n* = 5
SVL	36.8	36.0	44.0
HL	12.5	11.1	14.2
HW	8.4	7.5	10.1
HH	4.4	4.3	5.2
OD	2.2	3.1	2.6
EE	4.3	4.7	4.7
IN	1.2	0.9	1.7
SE	5.5	4.7	6.3
TrunkL	14.7	14.7	16.6
HindLL	7.4	9.8	8.0
CL	7.0	8.0	8.2
FL	6.0	6.7	7.0
PCP	2 + 2	0 or 1 + 1	(3–5) + (3–5)
Lamellae 4th Toe	7 + (9–10)	(6–7) + (6)	(7–8) + (10–11)
Supralabials	9–11	9–11	7–9
Infralabials	7–8	7–8	7
Internasal	1 + 1	1 + 1	1 + 1
Subcaudals	Small irregular	Small irregular	Medium‐irregular

*Note:* Measurements in millimeters (mm). For abbreviations, see Section 2. For detailed measurements see Table S4.

*Rhoptropus* sp. Lobón‐Rovira et al. 2022: 306.

**Holotype**. MNCN 53501 (field number P4.238), adult female with incision in the abdominal region, collected in Bentiaba region, Namibe Province (−14.1250, 12.5361), Angola, on September 21, 2024 by JLR and PVP.

**Paratypes**. MNCN 53502 and MHNCUP/REP0988 (field number JLRZC0089–90, respectively), adult males, collected in Baba region, Namibe Province (−14.8354, 12.2296) on February 17, 2020 by JLR and PVP. FKH 0617 (field number P1.191), adult male, collected in Praia de Soba, Namibe Province (−14.7467, 12.2894) on August 10, 2021 by PVP. FKH 1446 (field number JLRZC0423), adult female, collected 20 km north of Moçamedes, Namibe Province (−15.0297, 12.2357) on February 6, 2025 by JLR and PVP.

**Diagnosis**. A small‐sized diurnal gecko (max. SVL < 39 mm), clearly belonging to the *R. barnardi* group, based on the presence of small and irregular subcaudal scales (Figure 3) and its phylogenetic placement (Figure 1). It has 9–11 supralabials and 7–8 infralabials. Dorsal pholidosis with small granular scales. Dorsum with light yellowish to gray background color, with soft orange‐rust reticulation pattern interspersed by small black and light cream to whitish spots extending from head to tail tip. The mental scale is rectangular, twice as long as it is wide, and followed by a uniform row of chin shields. The nostrils are surrounded by three tubercular nasal scales. Nasal scales can be in direct contact or separated by one or two internasal scales. Ventral pholidosis with larger, flattened, juxtaposed scales. Males with four precloacal pores, separated into two groups by two or three enlarged poreless scales (Figure 4, Table S4). Digits elongated, with 16–18 (10 + 7) lamellae under the fourth toe.

**Comparative diagnosis**. This species can easily be distinguished from other *Rhoptropus* based on its smaller size (max. SVL < 39 mm vs. > 46.00 mm [fide Bauer and Good 1996; Kuhn 2016]) and the characteristic dorsal coloration stated above. Additionally, it differs from *R. boultoni* , the *R. benguellensis* group, *R. montanus* , *R. nivimontanus*, and *R. taeniostictus* by the lack of enlarged subcaudals on original tails. From *R. boultoni* , *R. benguellensis* , and *R. montanus* , it differs by having black spots on the dorsum.

It can also be distinguished from other members of the *R. barnardi* group as follows: from *R. barnardi* and *R*. aff. *barnardi* by having fewer precloacal pores, which are separated into two groups by enlarged poreless scales (4 [2 + 2] in *R. minimus* sp. nov. vs. 6–9 arranged in a continuous row in the other two species or larger number of pores separated by one or two enlarged scutes; Figure 3). It can also be distinguished from these two species based on dorsal coloration (Figure 4). Thus, *Rhoptropus minimus* sp. nov. presents a “granitic” appearance of the dorsum, comprising a light background color with a soft orange‐rust reticulation pattern evenly distributed across the dorsum, interspersed with small black and light cream to whitish spots. In contrast, the other two species exhibit a light brown to gray dorsum with an orange‐rust reticulated pattern surrounding large, light cream vertebral areas, interspersed with larger black spots and smaller light cream spots (Figure 3). This pattern is more variable in *R*. aff. *barnardi* but usually retains visibly lighter vertebral areas (Figure 3). In addition, it can be distinguished from these two species by having a more ground‐dwelling behavior versus a more rupicolous, and by lack of haplotype sharing in the nuclear markers and more than 10% in the *ND2* uncorrected p‐distance (Table 1). Finally, it can be distinguished from *R. biporosus* by its smaller size (max. SVL < 39 mm versus 50.5 mm [fide Bauer and Good 1996]), by having more precloacal pores (4 vs. 0–2 in *R. biporosus* ), and by possessing a more robust and stout body and limbs, in contrast to the slenderer body and limbs of *R. biporosus* .

**Description of the holotype** (Figure 5). Adult female, with a ventral incision for extraction of the liver for genetic analysis (Figure 5A) and tail full but regenerated. SVL 36.9, TL 25.8, HW 9.4, HH 4.2, and HL 13.0 mm.

Dorsal scales are small, granular, and juxtaposed, with the scales on the frontal region being twice the size of those on the rest of the body. The rostral scale is proportionally large, pointed, extending between the first nasal scales. The nostril is surrounded by three nasal scales that are raised to yield an inflated nostril rim. The nasal scales are separated by the rostral and two small internasal scales. There are 22 interorbital scales, 11 supralabial scales, and eight infralabial scales on the right and left sides. The mental scale is elongated, longer than it is wide. The first supralabial is slightly smaller than the mental but almost twice the size of the second supralabial. The labials are followed by a row of small chin shields that are equal in size. The gular scales gradually decrease in size until the level of the posterior margin of the eyes, beyond which they become distinctly smaller and uniform in size. There are two enlarged precloacal scales (without an opening) separated by a smaller scale, corresponding to precloacal pore‐bearing scales in males. The right fourth toe has seven terminal scansors, followed by 11 lamellae, while the left fourth toe is missing. The first segment of the tail is original and has the same pholidosis as the regenerated portion, which consists of small, irregularly sized subcaudal scales.

**Coloration**. *In life* (after capture): The dorsum has a gray background coloration, interspersed with small black and light white to cream dots from head to tail tip (Figure 4). In the holotype, the regenerated tail presents a uniform coloration, lacking any dots or ornamentation. The venter, gular region, and the underside of the tail and limbs have a uniform whitish to cream coloration, with only the tips of the fingers and toes being darker. *In preservative*: No evident change has been observed, with the dorsal pattern of the holotype remaining visible.

**Etymology**. The name *minimus* comes from the Latin adjective *minimus*, the superlative form of *parvus*, meaning “smallest” or “very small.” Since *Rhoptropus* is a masculine genus, as indicated by Peters (1869) in the original description, we use the masculine nominative form *minimus* to maintain grammatical agreement. Thus, the name refers to the characteristically small size of the species. The name refers to the characteristically small size of the species. As a common name, we suggest “Miniature Namib Day Gecko” in English and Osga‐diurna‐do‐Namibe miniatura in Portuguese.

**Distribution and natural history** (Figures 6 and 7). *Rhoptropus minimus* sp. nov. is an Angolan endemic, ground‐dwelling species known only from the coastal regions of Namibe Province. It is most abundant in the Baba and Praia do Soba regions in central Namibe Province, where it is primarily associated with gravel and sand plains in sedimentary areas. The species appears to extend northward to the Bentiaba region, from where the type locality has been designated, and southward to Salondjamba (Lobón‐Rovira et al. 2022), near the entrance of Iona National Park (Figure 6A). At the southern edge of its distribution, *R. minimus* sp. nov. appears to be replaced by another ground‐dwelling day gecko, *R. biporosus* (Figure 6A). However, there are no reports of sympatry between the two species, with *R. biporosus* being abundant in the gravel and sand plains of Iona NP from Espinheira southward to Cunene River (Lobón‐Rovira et al. 2022). Interestingly, while *R*. aff. *barnardi*, the closest member of the *R. barnardi* group, is the most widespread and abundant species in Namibe Province showing potential range overlap (Figure 6B), *R*. aff. *barnardi* seems to be absent from localities where *R. minimus* sp. nov. is present. This may be due to habitat preferences, as *R. minimus* sp. nov. is primarily found in more open areas (Figure 7A,B), where it moves quickly between small rocks and loose flakes on the ground, using them for shelter versus more rocky areas with large boulders for which *R*. aff. *barnardi* seems to have a preference (Figure 7C,D). However, in both the southernmost and northernmost areas, the species is also found in dry valley regions, where it remains active on the ground on the sand of dry riverbeds, supporting the hypothesis that these areas represent the most extreme parts of its distribution.

**FIGURE 8:**
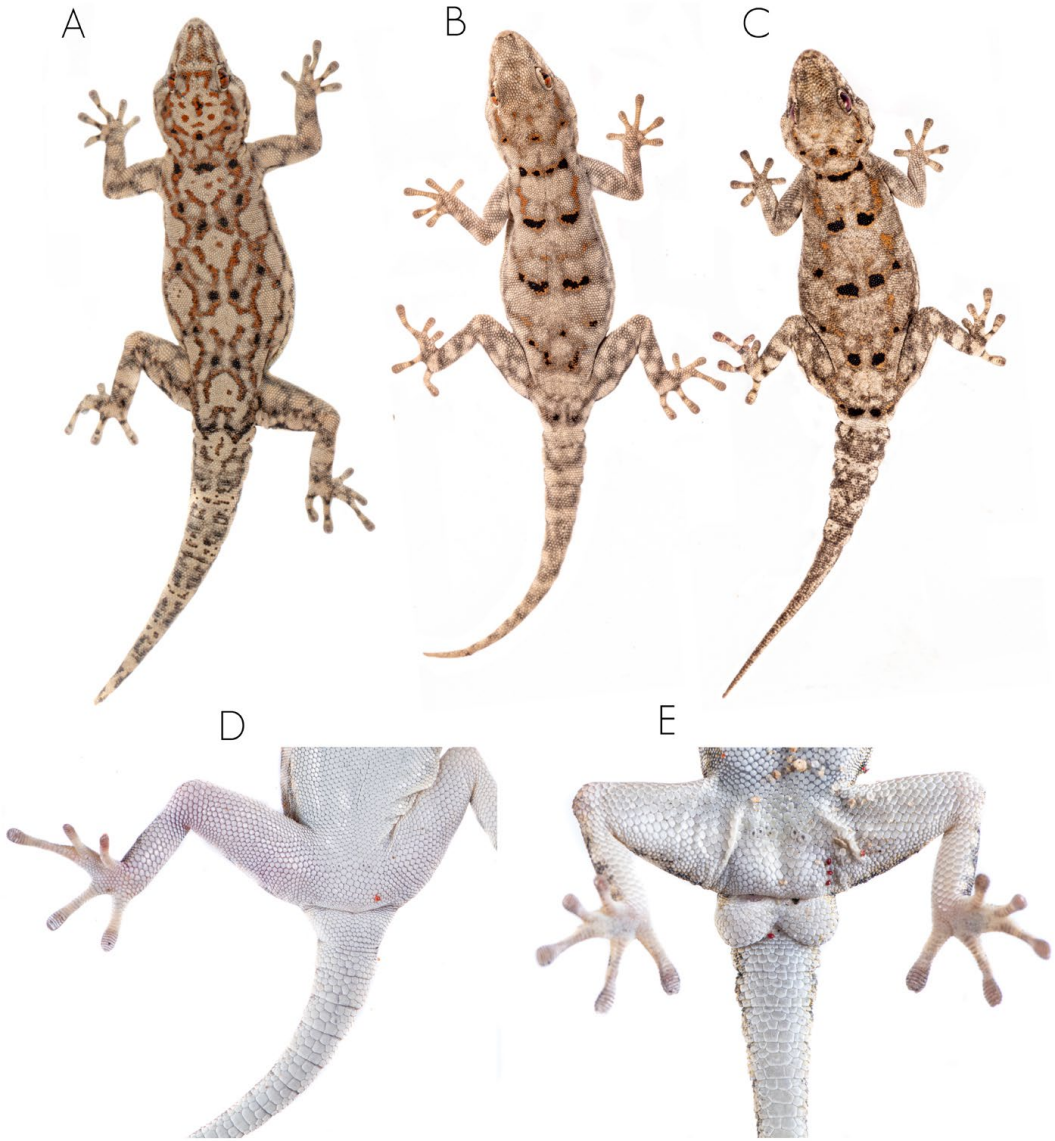
Unscaled photographs of the dorsal color pattern variation of the members of the *Rhoptropus taeniostictus* group in Angola. (A) *Rhoptropus taeniostictus* sensu stricto from Caraculo, Namibe Province, Angola. (B, C) *Rhoptropus megacellus* sp. nov. from Bentiaba, Namibe Province, Angola. (D, E) Detail of the cloacal region of *R. taeniostictus* sensu stricto and *R. megacellus* sp. nov., respectively, showing the cloacal pores arrangement and/or the subcaudal scales.

**FIGURE 9:**
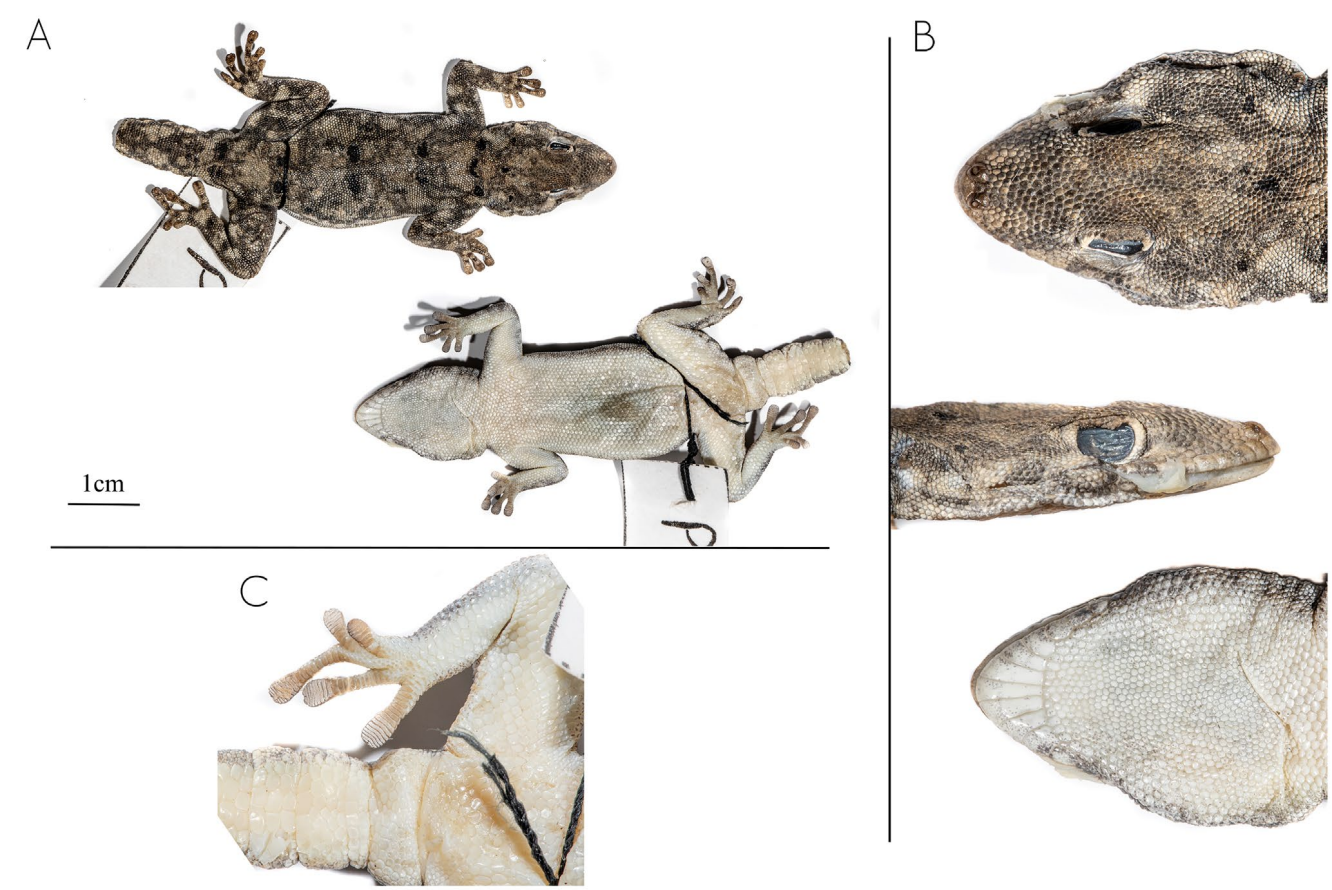
Holotype of *Rhoptropus megacellus* sp. nov. (MNCN53503; Field Number P4.323) from Bentiaba, Namibe Province, Angola. (A) Photographs of the full body in dorsal (above) and ventral (below) views. (B) Details of the head in dorsal, lateral and ventral views. (C) Detail of the feet and subcaudals.

**FIGURE 10:**
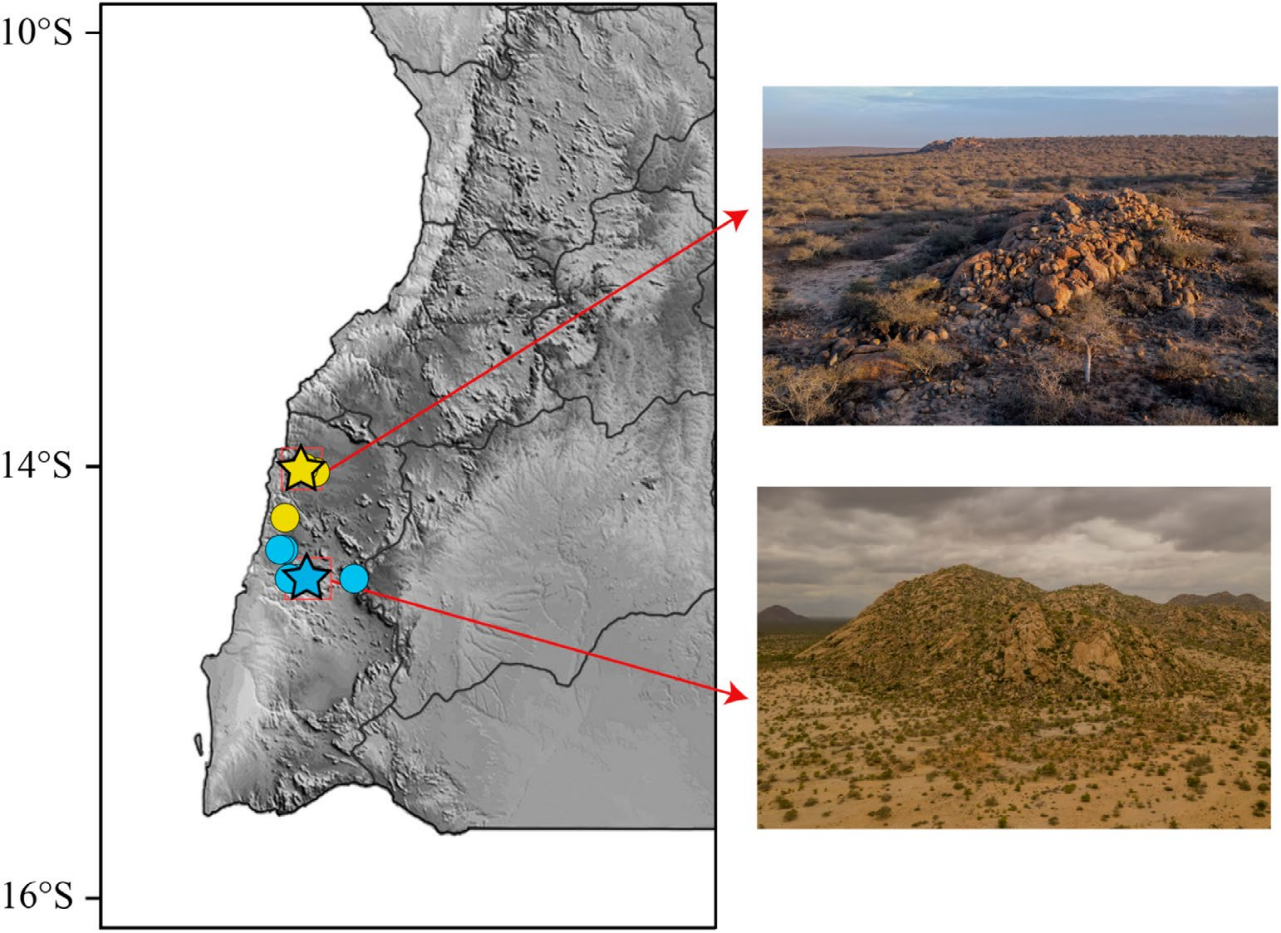
Distribution of the members of the *Rhoptropus taeniostictus* group in Angola. Distribution of *R. megacellus* sp. nov. (yellow) and *R. taeniostictus* sensu stricto (blue), highlighting the habitat at the type locality of the different species. Stars depict the type localities.

***Rhoptropus megocellus* sp. nov**.

https://zoobank.org/57A5FCD9‐1C82‐41B5‐BF12‐4B3942C44A4D

(Figures 8, 9, 10, Table 3 and Table S4)

**TABLE 3:** Means of the morphological (morphometric and meristic) characteristic of members of the *Rhoptropus benguellensis* and the *R. taeniostictus* groups.

Species	*R. megocellus* sp. nov.	*R. taeniostictus*	*R. crypticus* sp. nov.	*R. benguellensis*
	*n* = 4	*n* = 6	*n* = 4	*n* = 5
SVL	51.0	52.0	49.5	51.5
HL	15.5	15.4	15.0	15.3
HW	12.0	11.9	10.5	10.8
HH	4.3	5.2	5.5	5.1
OD	2.8	2.9	2.9	2.8
EE	5.7	5.5	4.6	4.6
IN	1.8	1.6	1.6	1.5
SE	7.3	7.0	6.9	6.7
TrunkL	21.1	20.1	18.1	22.4
HindLL	9.8	9.9	9.0	10.8
CL	9.6	9.9	9.0	8.7
FL	7.3	8.2	8.2	8.8
PCP	3 + 3	4 + 3	(2–3) + (2–3)	(3–4) + (3–4)
Lamellae 4th Toe	8 + (10–12)	(10–11) + (10–12)	(7–8) + (11–12)	(6–9) + (7–11)
Supralabials	9–11	9–11	9–11	9–11
Infralabials	7	7–8	7–8	7
Internasal	1	1	1 + 1	1 + 1
Subcaudals	Large paired	Large paired	Large single	Large single

*Note:* Measurements in millimeters (mm). For abbreviations, see Section 2. For detailed measurements see Table S4.

**Holotype**. MNCN 53503 (field number P4.323), adult male with half tail, collected in Bentiaba region, Namibe Province (−14.14130, 12.5790), Angola, on November 11, 2024 by PVP.

**Paratype**. MHNCUP/REP0989 (field number P4.321) and FKH 1421 (field number P4.322), adult females, and FKH 1424 (P4.329) adult male, with same collecting information as the holotype.

**Diagnosis**. A large sized day gecko (max. SVL 56.9 mm), clearly belonging to the *R. taeniostictus* group on the basis of the double series of transversely enlarged subcaudals (Figure 8) and phylogenetic results (Figure 1). It has 9–11 supralabials and 7 infralabials. Dorsal pholidosis with small granular scales. Dorsum with light to dark gray background color, with a series of paired large ocelli on its dorsum. The mental scale is rectangular, twice as long as it is wide, and followed by a uniform row of chin shields. The nostrils are surrounded by three tubercular nasal scales, separated by one large internasal. Ventral pholidosis with larger, flattened, juxtaposed scales. Males with six precloacal pores, separated into two groups by two enlarged poreless scales (Figure 8E). Digits elongated, 18–20 (8 + 10–12) lamellae under the fourth toe.

**Comparative diagnosis**. It differs from other large bodied *Rhoptropus*, namely *R. benguellensis* , *R. montanus* , *R. nivimontanus*, and *R. boultoni* , by having large black dorsal ocelli (Figure 8), absent in the other species. It differs also from these species by having large paired subcaudal scales versus a single series of enlarged subcaudal scales on original tails.

It differs from members of the *R. barnardi* group by having enlarged paired subcaudal scales on the original tail, whereas other species have small to medium irregularly distributed scales. Additionally, it has larger ocelli on the dorsum, arranged in groups of two, in contrast to the smaller black spots that are either irregularly distributed or arranged in a hexagonal pattern in other species. It can also be distinguished by the presence of a single large internasal scale, as opposed to two small internasals or no internasal scales.

Finally, it differs from its close relative, *R. taeniostictus* , by having larger dorsal ocelli with a less pronounced orange reticulated pattern on the dorsum. Additionally, it has fewer terminal scansors (8 vs. 10–11 in *R. taeniostictus* ).

**Description of the holotype** (Figure 9). Adult male, in good state of preservation, tongue, and posterior part of the tail removed for genetic investigations. SVL 56.9, HW 13.7, HH 4.4, and HL 17.9 mm.

Dorsal scales are small, granular, and juxtaposed, with the scales on the frontal region twice the size of those on the rest of the body. The rostral scale is proportionally large, pointed, extending between the first nasal scales. The nostril is surrounded by three nasal scales that are raised to yield an inflated nostril rim. The nasal scales on each side are separated by the rostral and one proportionally large (when compared to other *Rhoptropus*) internasal scale. There are 17 interorbital scales. There are ten supralabial scales and seven infralabial scales on the right and left sides. The mental scale is elongated, longer than wide. The first supralabial is slightly smaller than the mental but almost twice the size of the second supralabial. The labials are followed by a row of small chin shields that are smaller in the central region and larger laterally. The gular scales gradually decrease in size until the level of the posterior margin of the eyes, beyond which they become distinctly smaller and uniform in size. Ventral scales are granular and larger than gulars. There are six precloacal scales, in two groups of three scales separated by two poreless scales. The right fourth toe has eight terminal scansors, followed by 11 lamellae. The first segment of the tail has irregularly sized subcaudal scales; beyond this point, all segments feature distinct pairs of enlarged subcaudal scales.

**Coloration**. *In life* (after capture): Dorsum with light gray to brown background coloration, interspersed by an orange reticulation and five consecutive pairs of large ocelli from neck to tail (Figure 8). The first pair of large ocelli on the neck may be in contact, giving a collar‐like appearance (Figure 8C). Hindlimbs and tail with light cream spots. The venter, gular region, and the underside of the tail and limbs have a uniform whitish to cream coloration, with only the tips of the fingers and toes being darker. *In preservative*: The orange reticulation is absent, with only the large black ocelli remaining visible on the dorsum.

**Etymology**. The name “megocellus” is a noun in apposition derived from the Ancient Greek “mega”, meaning “large,” and the Latin “ocellus”, meaning “eye spot.” This name refers to the species' distinctive large ocelli. As a common name, we suggest “Large‐spotted Namib Day Gecko” in English and “Osga‐diurna‐de‐manchas‐grandes do Namib” in Portuguese.

**Distribution and natural history** (Figure 10). An Angolan endemic species, only known from the Bentiaba region and the granitic boulder region north of Chapeu Armado. Despite its very restricted distribution, the species appears to be abundant where it is present. Like its sister species, *R. taeniostictus* , this species is strictly rock‐dwelling, always found on large, flattened granitic boulders, where it can run rapidly to escape. It is frequently found hiding under exfoliated flakes on these flattened granitic surfaces. Despite having a similar ecological niche preference to its sister species, the two have not been found in sympatry. However, this species frequently occurs in sympatry with two other large rock‐dwelling *Rhoptropus* species, namely *R. boultoni* and *R. crypticus* sp. nov. However, these two species are usually found between large boulders on vertical surfaces, displaying some ecological niche segregation.

**FIGURE 11:**
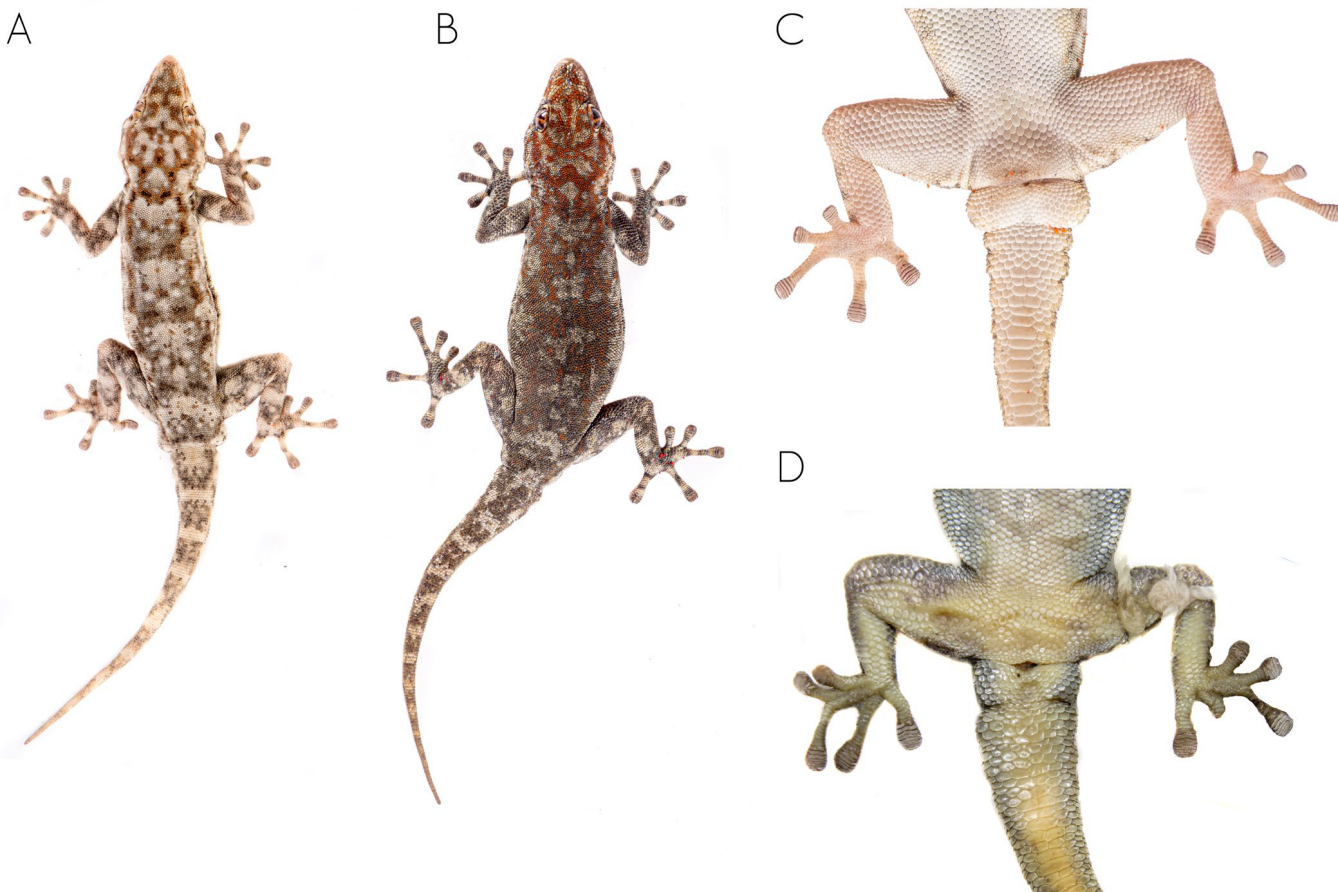
Unscaled photographs of the dorsal color pattern variation of the members of the *Rhoptropus benguellensis* group in Angola. (A) *R. crypticus* sp. nov. from Bentiaba, Namibe Province, Angola. (B) *R. benguellensis* from Cambau, Cuanza Sul Province. (C, D) Detail of the cloacal region of *R. crypticus* sp. nov. and *R. benguellensis* , respectively, showing the cloacal pores arrangement and the subcaudal scales.

**FIGURE 12:**
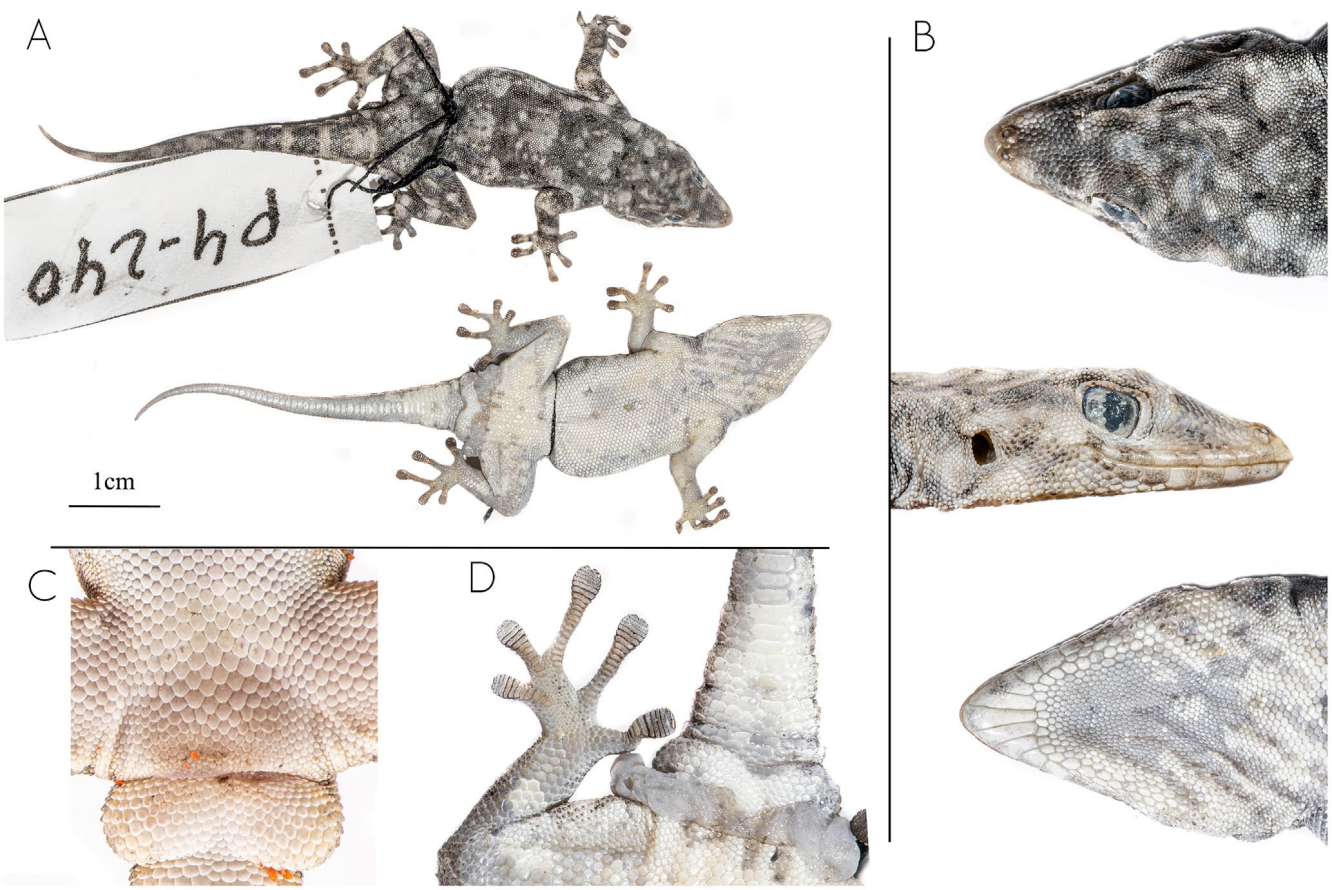
Holotype of *Rhoptropus crypticus* sp. nov. (MNCN53504; Field Number P4.240) from Bentiaba, Namibe Province, Angola. (A) Photographs of the full body in dorsal (above) and ventral (below) views. (B) Details of the head in dorsal, lateral and ventral views. (C) Detail of the cloacal region in live specimen. (D) Detail of the feet, hemipenal region and subcaudals.

**FIGURE 13:**
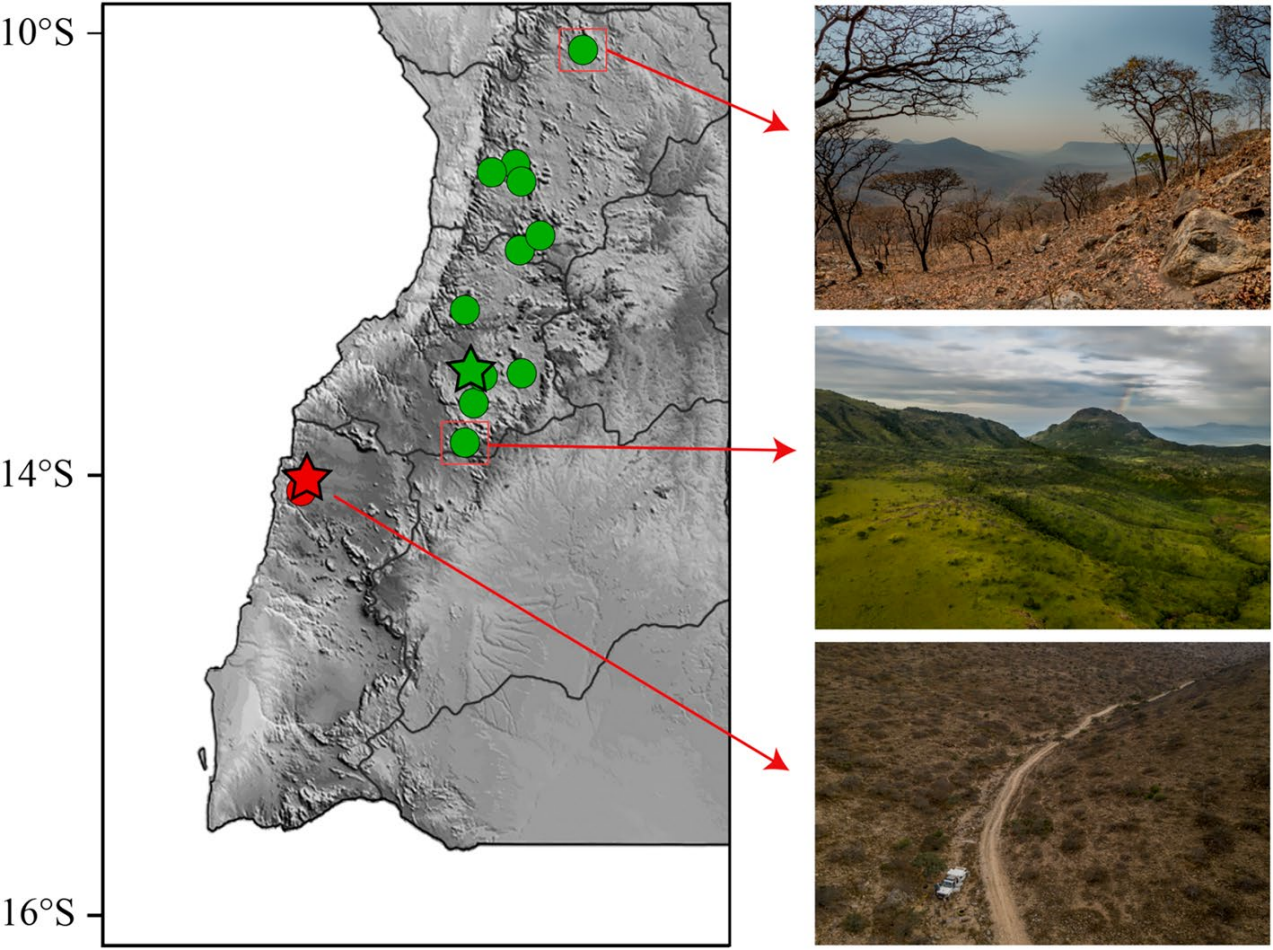
Distribution of *Rhoptropus benguellensis* (green) and *R. crypticus* sp. nov. (red), highlighting the differences of habitat across the geographic distribution of the species. Stars depict the type localities.

***Rhoptropus crypticus* sp. nov**.

https://zoobank.org/C99992DE‐649E‐4B91‐88E9‐E755F192629D

(Figures 11, 12, 13, Table 3 and Table S4)

**Holotype**. MNCN 53504 (field number P4.240), adult male with incision in the abdominal section, collected in Bentiaba region, Namibe Province (−14.1250, 12.5361), Angola, on September 21, 2024 by JLR and PVP.

**Paratypes**. MHNCUP/REP0990 & FKH 1466 (field number JLRZC0396, JLRZC0474, respectively) adult males, collected in Bentiaba region, Namibe Province, Angola, on February 5, 2025 by JLR and PVP. FKH 1442 (field number JLRZC0397) adult female, with the same collecting information as JLRZC0396 (see Table S1).

**Diagnosis**. A large sized day gecko (max. SVL 54.7 mm), clearly belonging to the *R. benguellensis* group (the *R. boultoni* group, fide Parrinha et al. 2025) by having unique series of large subcaudal scales on original tails (Figure 11). It has 9–11 supralabials and 7–8 infralabials. Dorsal pholidosis with small granular scales, with markedly orange reticulated coloration on the head which disappears toward the tail, being interspersed by medium sized black spots on the dorsum. The mental scale is rectangular, twice as long as wide, and is followed by a uniform row of chin shields. The nostrils are surrounded by three nasal scales that are raised to yield an inflated nostril rim, separated by two small internasals (Figure 12). Ventral pholidosis with larger, flattened, juxtaposed scales. Males with 4–6 precloacal pores in two groups of two separated by two or three enlarged poreless scales (Table 3). Digits elongated, 18–20 (7–8 + 11–13) lamellae under the fourth toe.

**Comparative diagnosis**. It can be easily distinguished from all members of the *R. barnardi* group by having enlarged subcaudal scales on the original tail, greater snout‐vent length (max. SVL 54.7 mm vs. < 47 mm in the *R. barnardi* group) and dorsal pattern (Figures 3 and 11). It can be distinguished from *R. biporosus* by having more precloacal pores in males (4–6 vs. 0–2 in *R. biporosus* ). From *R. barnardi* sensu stricto, it can be distinguished by having a different dorsal pattern and precloacal pores separated in groups of two by enlarged scutes (frequently in a continuous row in *R. barnardi* sensu stricto).

It differs from members of the *R. taeniostictus* group by having a single series of enlarged subcaudal scales on the original tail (versus paired enlarged subcaudals), the presence of two small internasals (vs. a single enlarged internasal in the *R. taeniostictus* group) and by having a distinctive dorsal pattern (Figures 8 and 11).

Finally, it can be easily distinguished from all other members of the *R. boultoni* group (fide Parrinha et al. 2025, namely *R. montanus* , *R. nivimontanus*, *R. boultoni* , and *R. benguellensis* sensu stricto), by the presence of black spots on the dorsum, which are absent in all other members of the group.

**Description of the holotype** (Figure 12). Adult male, in good state of preservation, tongue removed for genetic investigations and hemipenes inverted. SVL 43.2, TL 42.9, HW 10.2, HH 4.7, and HL 14.1 mm.

Dorsal scales are small, granular, and juxtaposed, with the scales on the frontal region twice the size of those on the rest of the body. The rostral scale is proportionally large, pointed, and extends between the anteriormost nasal scales. The nostril is surrounded by three nasal scales that are raised to yield an inflated nostril rim. The nasal scales are separated by the rostral and two small internasal scales. There are 17 interorbital scales. Nine supralabial scales and eight infralabial scales are present on the right and left sides. The mental scale is elongated, longer than it is wide. The first supralabial is slightly smaller than the mental but almost twice the size of the second supralabial. The labials are followed by a row of small irregular chin shields. The gular scales gradually decrease in size until the level of the posterior margin of the eyes, beyond which they become distinctly smaller and uniform in size. Ventral scales are granular and larger than gulars. There are four precloacal scales in two groups of two separated by three enlarged poreless scales. The right fourth toe has eight terminal scansors, followed by 11 lamellae. The first two segments of the tail have irregularly sized subcaudal scales; beyond this point, all segments are characterized by distinctly enlarged subcaudal scales.

**Coloration**. *In life* (after capture): Dorsum with light gray to brown background coloration, interspersed by an orange reticulation on the head that turns to gray on the second half of the body. Orange and gray reticulations with a series of black ocelli and white speckles from head to tail (Figure 11). Hindlimbs with light cream spots and tail with a series of light gray and light cream crossbands. The venter, gular region, and the underside of the tail and limbs have a uniform whitish to cream coloration. *In preservative*: The orange reticulation is absent, with only the large ocelli and the lighter speckles remaining visible on the dorsum.

**Etymology**. The name “crypticus” is derived from the Latin adjective “crypticus”, meaning “hidden” or “concealed.” It is used here in the masculine form to agree with the masculine grammatical gender of the genus *Rhoptropus*. Thus, “crypticus” refers to the species' cryptic or camouflaged nature. As a common name, we suggest “Cryptic Namib Day Gecko” in English and “Osga‐diurna‐do Namib criptica” in Portuguese.

**Distribution and natural history** (Figure 12). An Angolan endemic species, *Rhoptropus crypticus* sp. nov. is only known from a very restricted area in the Bentiaba region, located in the northernmost part of Namibe Province. Although this region is poorly explored, ecological niches similar to the one where this species has been found are quite rare in the area, suggesting high isolation of the species (Figure 12). The species is strictly rock dwelling, frequently found moving among vertical surfaces of large boulders in the spiny arid savanna. The species appears to have clear ecological segregation from its closely related species, *R. benguellensis* . While *R. crypticus* sp. nov. is confined to the arid coastal region of Angola, *R. benguellensis* is widely distributed across the Angolan escarpment and the central highlands of Benguela and Cuanza Sul provinces (Figure 12). On the other hand, *R. crypticus* sp. nov. has been found in close sympatry and syntopy with two other large rock‐dwelling *Rhoptropus* species, namely *R. boultoni* and *R. megocellus* sp. nov. While the species shows some ecological segregation with respect to *R. megocellus* sp. *nov*. (see above), we found no evidence of ecological segregation from *R. boultoni* . Despite its very restricted distribution, the species appears to be locally abundant.

## 4 Discussion

In this work we described three endemic Angolan geckos, which contributes to the ongoing increase in knowledge of Angolan reptile diversity. With the addition of these two species, the number of described Angolan geckos rises to 55, with one additional candidate species (*R*. aff. *barnardi*) and some species complexes that remain unresolved (e.g., *Pachydactylus oreophilus* complex, Bauer et al. in prep.; *P. punctatus* complex, Bauer et al. in prep.). As a result of this work, the genus *Rhoptropus* is now recognized to include 13 described species and two additional candidate species (Parrinha et al. in prep), including seven strictly endemic Angolan species (i.e., *R. montanus* , *R. nivimontanus*, *R. benguellensis* , *R. cypticus* sp. nov., *R. minimus* sp. nov., *R. taeniostictus* , *R. megocellus* sp. nov.) and 12 species recorded in its territory. This suggests that Angola represents the primary diversification center for this genus.

### 4.1 The *Rhoptropus barnardi* Group

In Angola, only two members of the *Rhoptropus barnardi* group have been described so far (Branch et al. 2019). Nevertheless, Kuhn (2016) identified a third putative candidate new species (*R*. aff. *barnardi*), which was morphologically more similar to *R. barnardi* sensu stricto but genetically closer to *R. biporosus* . Our work supports the recognition of *R*. aff. *barnardi* as a candidate new species and we describe a new species within this group, *R. minimus* sp. nov. These findings resolve the *R. barnardi* group into four species within Angola's territory, showing some biogeographic and ecological niche segregation between sister taxa.

It must be noted that Lobón‐Rovira et al. (2022) reported an undescribed taxon (*Rhoptropus* sp.) from Salondjamba, at the entrance of Iona National Park, as a member of this group, provisionally ascribed to *R*. aff. *barnardi*. However, the taxonomic identification was based on *16S* barcoding analysis, primarily through comparison with previously available genetic information and the material included in that study (Lobón‐Rovira et al. 2022). Nevertheless, thanks to wider genetic and morphological information, this material proved not to be *R*. aff. *barnardi* but *R. minimus* sp. nov., described here.

As mentioned above, these four putative species exhibit biogeographic and ecological segregation. The case of *R*. aff. *barnardi* is particularly remarkable, as despite its wide distribution in Angola, this species has never been reported in sympatry with its closest relative, *R. minimus* sp. nov. On the other hand, *R. barnardi* and *R. biporosus* are frequently found in sympatry at Iona National Park (Lobón‐Rovira et al. 2022). Nevertheless, these two species present a marked ecological segregation. While *R. barnardi* is consistently found on large boulders, exhibiting a clear rupicolous lifestyle, *R. biporosus* is often found on the ground, moving across open areas between different outcrops. It should be noted that Kuhn (2016) and Ceríaco et al. (2016) reported *R*. aff. *barnardi* from Iona NP. However, our wide sampling effort does not allow us to corroborate the presence of the species south of Giraul, a few kilometers north of Moçamedes, Namibe Province. Therefore, our results suggest that this species is isolated from *R. barnardi* sensu stricto, which is widely distributed in Iona NP and southwestern Cunene Province at Calueque, from where the species was originally described.

Finally, the second lineage, referred to as *R*. aff. *barnardi* 2 in the molecular analysis, might also represent a candidate new species, based solely on the phylogenetic results. Notwithstanding, while most *Rhoptropus* species seem to diversify between the Mid‐Miocene and the Late Miocene [~5–20 Mys], some members of the *R. barnardi* group (namely, *R*. aff. *barnardi* 1 + *R. minimus* sp. nov. + *R*. aff. *barnardi* 2), are estimated to have speciated during between the Late Miocene and the Pliocene [~2–5.8 Mys]. Then, based on the bioclimate hypothesized as a main driving force in speciation of southern escarpment taxa as reported in other groups of reptiles (Conradie et al. 2022; Lobón‐Rovira et al. in prep), the bioclimatic events in the southern escarpment in the Pliocene could be invoked to explain the speciation of species on the *R. barnardi* group. However, both the morphological comparison and the median‐joining nuclear network suggest caution in interpreting *R*. aff. *barnardi* 2 as a distinct taxon. Morphologically, this lineage does not exhibit any distinct diagnostic characters differentiating it from *R*. aff. *barnardi* 1, and the *RAG‐1* haplotype network shows that this mitochondrial lineage shares all the haplotypes with *R*. aff. *barnardi* 1. In addition, both lineges are found in the highlands of the southern escarpment, at Tundavala and Leba Pass (Figure 5), suggesting some biogeographic overlap. This pattern has been observed in other groups of reptiles (e.g., *Afroedura*, Conradie et al. 2022; and *Cordylus*, Lobón‐Rovira et al. in prep), challenging the taxonomic stability of some species, such as *C. namakuiyus* , due to mito‐nuclear incongruence that is currently being explored (Lobón‐Rovira et al. in prep). Consequently, we recommend caution in taking further taxonomic actions within this group and suggest using next‐generation sequencing techniques to explore potential evolutionary traces of introgression and/or ancient polymorphisms.

### 4.2 The *Rhoptropus taeniostictus* Group

*Rhoptropus taeniostictus* was originally described based on a young female from “km 60 in the road between Moçamedes and Sá de Bandeira (= Lubango)” (Laurent 1964). This agrees with the locality of the village of Caraculo. from which our material was collected, as was the material reported by Kuhn (2016). Nevertheless, our results suggest that this species is distributed across the arid region northward to Mariquita. As in other *Rhoptropus* groups, we found limitedgeographic isolation between the two members of this group, with *R. megocellus* sp. nov. present at Chapeu Armado, ~40 km north of the northernmost record of *R. taeniostictus* sensu stricto, suggesting that the two species might be in sympatry in nearby areas, due to the continuity of the habitat in that region. Nevertheless, we found no evidence of gene flow between the two species, based on the lack of shared haplotypes. Finally, our results support the importance of the bioclimatic shift in northern Benguela Province during the Miocene. This period has been proposed as a key time for promoting speciation in Africa (Plana 2004), but its effect on the coastal region of Angola remains poorly understood.

### 4.3 The *Rhoptropus* *b* *enguellensis* Group

*Rhoptropus benguellensis* was originally described as a subspecies of *R. boultoni* (Mertens 1938), which was later elevated to full species status (Marques et al. 2018). It is more closely related phylogenetically to the *R. barnardi* group than to *R. boultoni* (Kuhn 2016; Heinicke et al. 2017; Parrinha et al. 2025; this work). This species is an endemic Angolan species distributed only in the Angolan escarpment savannas and woodlands (Ecoregion 6, Huntley 2023). Therefore, the results of this work support the previously published hypothesis but also shed light on the evolutionary history of the group, adding a link between the coastal region and northern Angolan escarpment. Thus, *R. crypticus* sp. nov. represents the coastal representative of the *R. benguellensis* group. In addition, this new species has an appearance mixing features of other *Rhoptropus*. It has the characteristic coloration of *R. benguellensis* and other species from the highlands and the northern escarpment (i.e., *R. montanus* and *R. nivimontanus*), which consist of an orange reticulated pattern (or more yellowish in *R. nivimontanus*) lacking any black spotted dorsal pattern on the body (Kuhn 2016; Parrinha et al. 2025). In addition, it has a dorsal pattern similar to that found in most coastal species. This pattern consists of an orange reticulated design on the dorsum, interspersed with light cream and black spots, as observed in *R. taeniostictus* and all members of the *R. barnardi* group.

### 4.4 Biogeography of Angolan Namib Day Geckos

To conclude, the results of this work contribute to resolving the biogeographic pattern of this still poorly understood group of geckos. The results support a main split between the central highlands of Angola (including the northern escarpment) and the coastal arid region, in agreement with other groups of reptiles like *Hemidactylus* (Lobón‐Rovira et al. 2021), *Afroedura* (Branch et al. 2021; Conradie et al. 2022) or *Cordylus* (Bates et al. 2023), which correspond mainly to a diversification between species during the Late Miocene. The case of the *R. barnardi* group on the southern escarpment of Angola is noteworthy. It follows the same biogeographic pattern found in other reptile groups (*Afroedura*, Conradie et al. 2022; *Cordylus*, Bates et al. 2023) presumably with introgression between coastal and adjacent highland forms, which requires further investigation. Nevertheless, the results of the time‐calibrated tree suggest that the *R. barnardi* group has experienced a second speciation process during the Late Miocene–Pliocene [2.5–5.8 Mys] in Angola.

In addition, while the northern Namibe Province region has been overlooked for herpetological studies for centuries, this region is currently being highlighted as an important center of endemism and diversification, especially for geckos (Lobón‐Rovira 2023). This work underscores the importance of the Bentiaba region and the sedimentary coastal region of Namibe as a potential center of endemism, probably driven by bioclimatic episodes in that region during the Late Miocene [4.5–10 Mys] that promoted isolation and speciation in this region, probably during events of expansion and regression on the northern rim of the Namib desert. Therefore, it can be expected that this region may host more overlooked undescribed taxa. This supports the importance of continuing fieldwork in this still poorly explored area to shed light on its true diversity, but also to identify the main evolutionary forces driving speciation and diversification in western southern Africa, and thereby develop informed conservation plans in this region.

## Author Contributions

**Javier Lobón‐Rovira:** conceptualization (lead), data curation (lead), formal analysis (lead), investigation (lead), methodology (lead), writing – original draft (lead). **Matthew P. Heinicke:** investigation (equal), methodology (equal). **Aaron M. Bauer:** supervision (equal), writing – review and editing (equal). **Werner Conradie:** resources (equal), writing – review and editing (equal). **Pedro Vaz Pinto:** conceptualization (equal), supervision (equal).

## Conflicts of Interest

The authors declare no conflicts of interest.

## Supporting information


**Figure S1.** Maximum likelihood (ML) phylogenetic tree for Angolan *Rhoptropus* based on *16S* gene using the most complete *Rhoptropus* dataset.

**Figure S2.** Maximum likelihood (ML) phylogenetic tree for Angolan *Rhoptropus* based on *ND2* gene using the most complete *Rhoptropus* dataset.

**Figure S3.** Time‐calibrated species‐level Bayesian phylogeny among Namib Day Geckos (*Rhoptropus*).

**Table S1.** List of material used for the phylogenetic analyses, including information on their field number, catalog number, country, localities, decimal geographic coordinates, and GenBank ascension numbers.

**Table S2.** Primers and PCR protocols used to generate sequences for the study.

**Table S3.** Genbank accession numbers of sequences used in Timetree analysis.

**Table S4.** *Rhoptropus* measurements used for the morphometric comparison.

## Data Availability

All the required data are uploaded as Supporting Information. Supporting Information is available at Ecology and Evolution Journal online. New generated genetic data (PV685180–PV685332; PV686014–PV686103; PV699739–PV699775) are available in GenBank (https://www.ncbi.nlm.nih.gov/genbank/) as specified in Tables S1 and S3.
