# Supplementary figures and images for: Three New Endemic Species of Namib Day Geckos (Gekkonidae: Rhoptropus) From the Namibe Province, Angola
Source: Ecol Evol. 2025 Jun 27;15(7):e71609. doi: 10.1002/ece3.71609 (PMC12204721; doi:10.1002/ece3.71609)

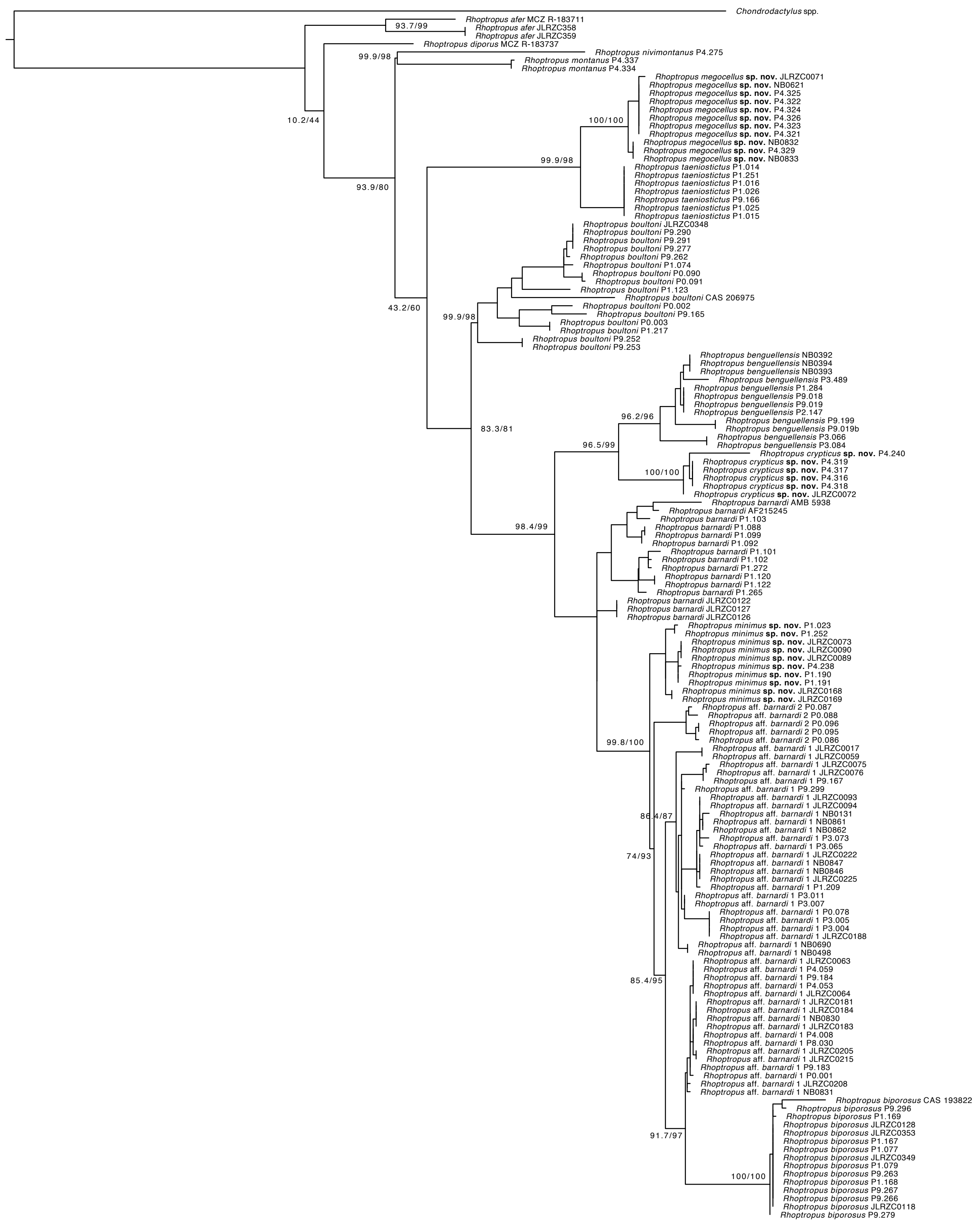

Supplement: Supplementary file 1 — Figure S1. Maximum likelihood (ML) phylogenetic tree for Angolan Rhoptropus based on 16S gene using the most complete Rhoptropus dataset. [file ECE3-15-e71609-s006.pdf]

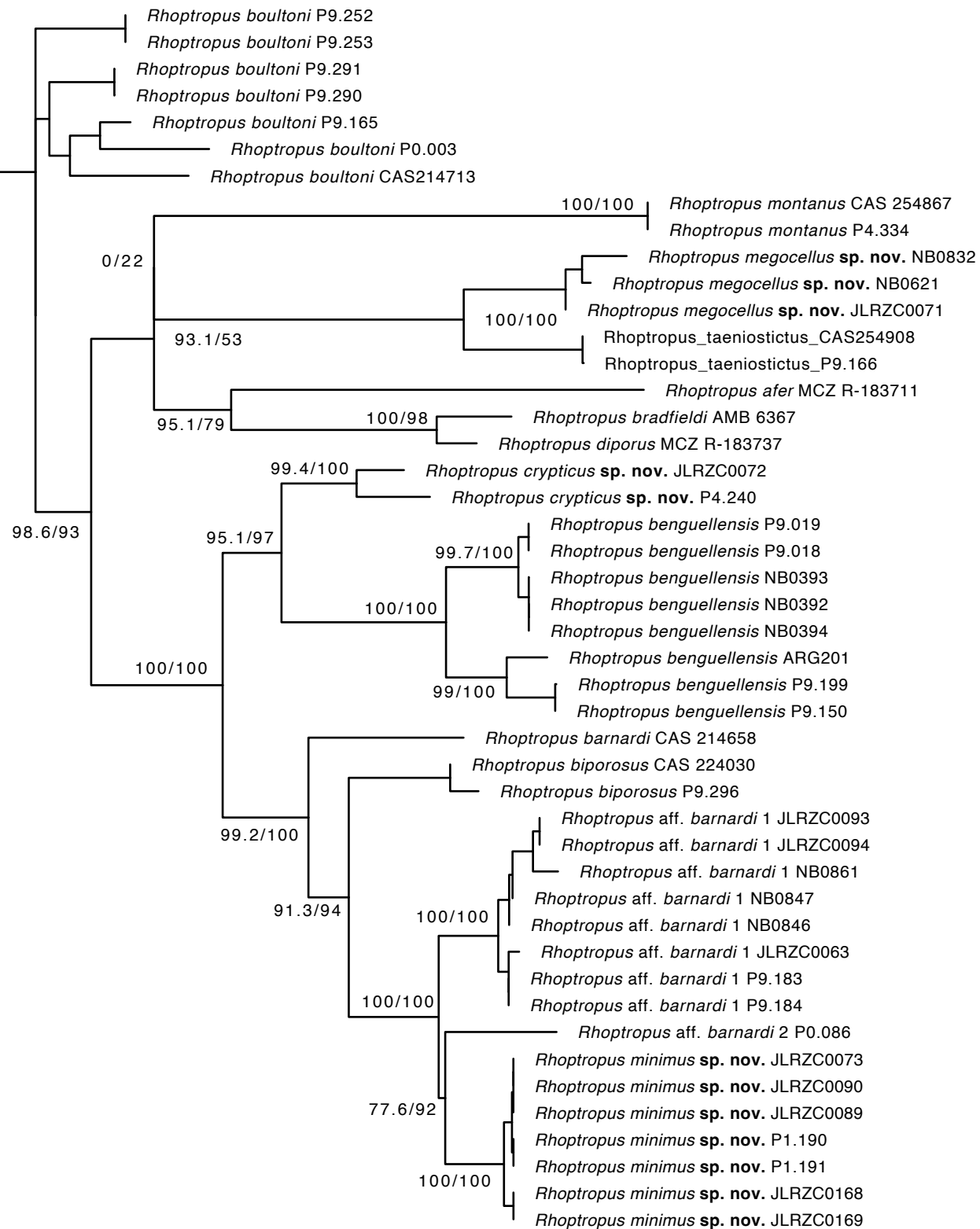

Supplement: Supplementary file 2 — Figure S2. Maximum likelihood (ML) phylogenetic tree for Angolan Rhoptropus based on ND2 gene using the most complete Rhoptropus dataset. [file ECE3-15-e71609-s004.pdf]

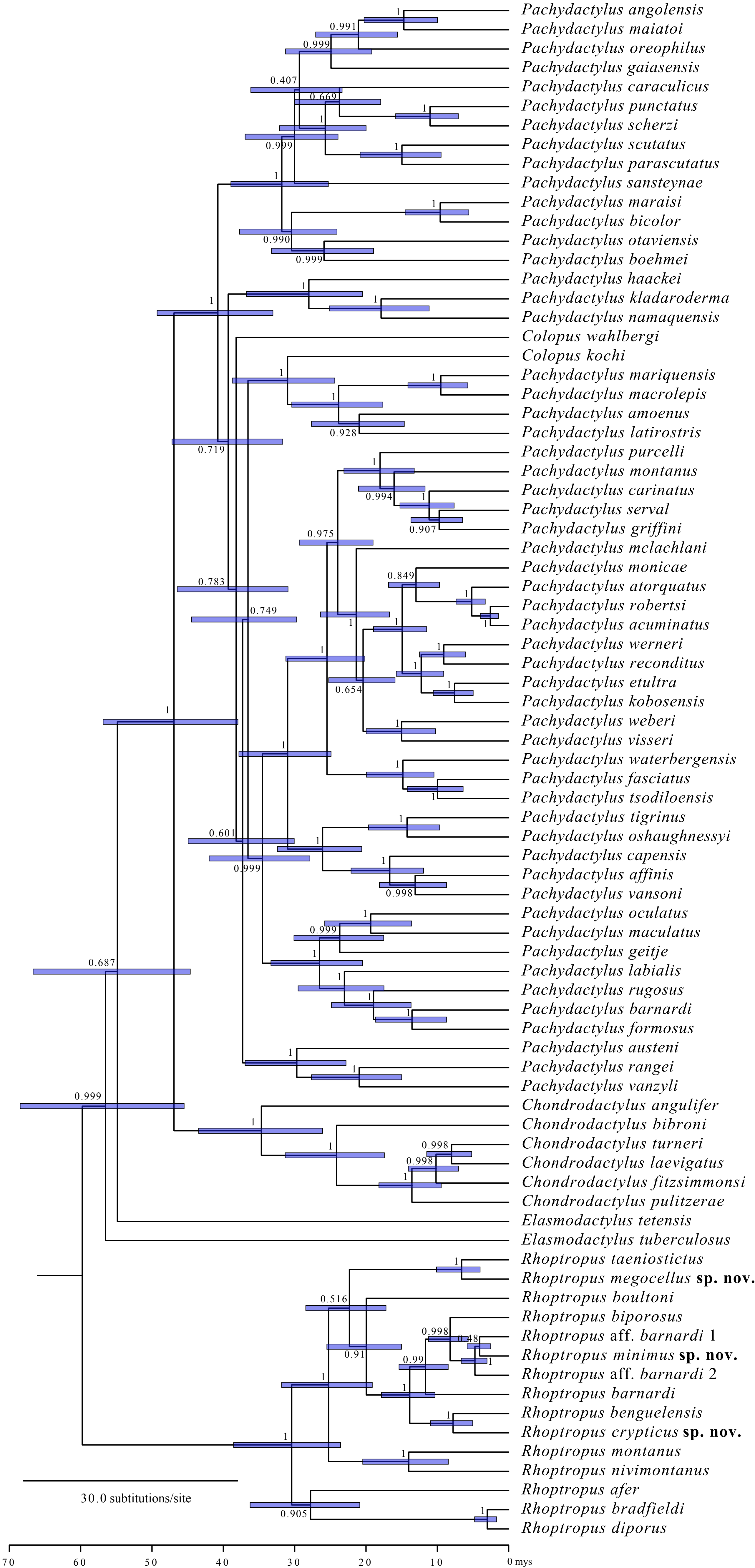

Supplement: Supplementary file 3 — Figure S3. Time‐calibrated species‐level Bayesian phylogeny among Namib Day Geckos (Rhoptropus). [file ECE3-15-e71609-s001.pdf]
